# Dairy intake, plasma metabolome, and risk of type 2 diabetes in a population-based cohort

**DOI:** 10.1016/j.ajcnut.2025.02.023

**Published:** 2025-04-03

**Authors:** Shunming Zhang, Suzanne Janzi, Yufeng Du, J Gustav Smith, Lu Qi, Yan Borné, Emily Sonestedt

**Affiliations:** 1 School of Public Health, Xi’an Jiaotong University Health Science Center, Xi’an, Shaanxi, China; 2 Nutritional Epidemiology, Department of Clinical Sciences Malmö, Lund University, Malmö, Sweden; 3 Department of Cardiology, Clinical Sciences, Lund University and Skåne University Hospital, Lund, Sweden; 4 Wallenberg Center for Molecular Medicine and Lund University Diabetes Center, Lund University, Lund, Sweden; 5 Department of Molecular and Clinical Medicine, Institute of Medicine, Gothenburg University and Sahlgrenska University Hospital, Gothenburg, Sweden; 6 Science for Life Laboratory, Gothenburg University, Gothenburg, Sweden; 7 Department of Epidemiology, School of Public Health and Tropical Medicine, Tulane University, New Orleans, LA, United States; 8 Department of Nutrition, Harvard T.H. Chan School of Public Health, Boston, MA, United States; 9 Department of Food and Meal Science, Faculty of Natural Science, Kristianstad University, Kristianstad, Sweden

**Keywords:** dairy, milk, yogurt, cheese, cream, butter, metabolomics, metabolites, type 2 diabetes

## Abstract

**Background:**

Whether dairy intake is related to type 2 diabetes (T2D) remains unclear, as does potential metabolic mechanisms for this association.

**Objectives:**

We aimed to examine the association between high dairy intake and risk of T2D and identify plasma metabolites reflecting dairy intake.

**Methods:**

This prospective cohort study included 26,461 Swedish individuals recruited between 1991 and 1996 and followed up until 31 December, 2020, with available data on dairy intake at baseline and linked registers. Plasma metabolites were measured in a subsample (*n* = 893) using mass spectrometry. Associations of dairy intake with risk of T2D were assessed using Cox proportional hazards models, with results presented as hazard ratios (HRs) and 95% confidence intervals (CIs).

**Results:**

A total of 4552 new-onset incident T2D cases were documented during a median follow-up of 24.3 y. Increased risk of T2D was observed among participants consuming high nonfermented milk (>1000 g/d compared with <200 g/d; HR: 1.40; 95% CI: 1.12, 1.74) and cheese (>100 g/d compared with <20 g/d; HR: 1.23; 95% CI: 1.07, 1.41), although decreased risk of T2D was observed among those with high fermented milk (>300 g/d compared with 0 g/d; HR: 0.88; 95% CI: 0.74, 1.03), cream (>50 g/d compared with <10 g/d; HR: 0.77; 95% CI: 0.64, 0.92), and butter (>50 g/d compared with 0 g/d; HR: 0.82; 95% CI: 0.71, 0.94). Such associations were slightly attenuated after additional adjustment for BMI. In addition, we identified metabolite profiles for nonfermented milk (*n* = 45), fermented milk (*n* = 48), cheese (*n* = 12), cream (*n* = 27), and butter (*n* = 46); no overlap between metabolites was found.

**Conclusions:**

In this cohort of Swedish adults, high intakes of nonfermented milk and cheese are positively associated with risk of T2D, although high intakes of fermented milk, cream, and butter are inversely associated. Metabolomics provides novel insights into understanding the metabolic pathways of these associations.

## Introduction

Diabetes is a growing global health challenge, with 537 million adults affected in 2021, projected to reach 783 million by 2045, and type 2 diabetes (T2D) accounting for >90% of cases [1]. Therefore, identifying potentially modifiable risk factors including diet for T2D is of major importance to public health interest.

Dairy products are widely consumed and have been associated in meta-analyses with risk of T2D but with inconsistent results [[2], [3], [4], [5]]. In particular, dairy products are a diverse food group, including nonfermented (e.g., milk) and fermented products (e.g., yogurt and cheese) as well as cream and butter. Owing to composition and processing differences, different dairy products could have variable effects on T2D. For example, the inverse association of T2D with milk intake was less robust than that with yogurt intake, and suggestive evidence indicated neutral associations or even an elevated risk associated with higher milk intake [2,4]. In addition, whether very high dairy intake is related to T2D remains unclear. We previously found inverse associations across quintiles for cream and butter but positive associations with nonfermented milk during 14 y of follow-up [6]. However, studies examining risk with very high intake levels (i.e., more than 1 L of milk or 100 g of cheese per day) are lacking due to a lack of data among populations with high dairy consumption. Therefore, additional studies of prospective cohorts with high-quality dietary data from populations with wide consumption ranges are needed to investigate dairy products separately for T2D. In Sweden, dairy intake is among the highest worldwide, and various dairy products are consumed in regular diets [7], which offers an exceptional opportunity to investigate the association between different types of dairy products and risk of T2D with a focus on more extreme intakes.

Metabolites are small molecules (e.g., amino acids and lipids) that represent intermediate or end products of metabolic reactions. Their levels can be influenced by diet and thus can be used to assess the metabolic responses to individual food intakes [8,9], or as markers of dietary intake [10,11]. Therefore, integrating metabolomics into traditional nutritional epidemiology studies can provide novel insights into the molecular mechanisms linking dairy products and T2D [12]. To our knowledge, only 2 studies have so far identified the plasma metabolites associated with dairy consumption and evaluated their associations with risk of T2D [13,14]. However, the 2 studies included total dairy (low-fat and high-fat dairy), milk, yogurt, cheese, and butter, without considering cream. In addition, limited metabolites (<400) were analyzed in the 2 studies. Therefore, additional untargeted metabolomics approaches with broader coverage of metabolites and dairy products including measurement on cream are needed.

In this study, we investigated the association between dairy intake (including nonfermented milk, fermented milk, cheese, cream, and butter) and risk of T2D, especially risk associated with very high intakes. We hypothesized that different dairy products may have heterogeneous effects on risk of T2D. Furthermore, we identified the plasma metabolites associated with dairy intake and assessed whether the metabolite profiles related to dairy products were specific to intakes of dairy products and other foods.

## Methods

### Study population

This study was based on the Malmö Diet and Cancer Study (MDCS), launched between 1991 and 1996 [15,16]. Participants were recruited from people living in Malmö, a city in southern Sweden, born during the period from 1923 to 1950. Upon enrollment, all participants were invited to complete a set of questionnaires about their dietary intakes, lifestyle factors, health status, and sociodemographic characteristics. Of the 74,318 invited participants, 30,446 individuals took part in the baseline examination. Every second participant who entered the MDCS between November 1991 and February 1994 was invited to take part in the Malmö Diet and Cancer-Cardiovascular Cohort (MDC-CC). In the MDC-CC, baseline fasting plasma samples were collected. Metabolomics profiling was conducted in a random subset of the MDC-CC participants (*n* = 928). The study was conducted according to the tenets of the Declaration of Helsinki guidelines and was approved by the Ethical Committee at the Medical Faculty at Lund University (approval number: LU 51-90). All participants signed written informed consent forms before participating in this study.

In this study, we excluded participants with missing data on dairy intake (*n* = 2212), those with diabetes at baseline based on linkage data as well as self-reported diabetes diagnosis/medication use (*n* = 1272), and those with missing covariates (*n* = 501), including missing BMI (*n* = 41), physical activity (*n* = 308), smoking (*n* = 7), education (*n* = 52), and a family history of diabetes (*n* = 93). The final analytic cohort for dairy intake and T2D included 26,461 individuals. Based on the principle of 10 outcome events per variable [17], this sample size was large enough to provide adequate statistical power. To identify metabolites related to dairy intake, we restricted the analytic sample to participants with data on metabolomics and dairy intake (*n* = 893). The sample size was determined based on the available resources [18]. Supplemental Figure 1 outlines the study design. The article was prepared following the STROBE guidelines [19] and the STROBE-nut guidelines [20].

### Assessment of dairy products

Usual food intake was assessed at baseline using a modified diet history method combining a 7-d food diary, a 168-item semiquantitative food frequency questionnaire (FFQ), and a 45–60 min dietary interview [21,22]. In the food diary, participants were asked to record their food intakes (usually cooked/main meals), cold beverages, and dietary supplements; the amount of dairy products in the cooked meals was collected. The FFQ collected information on foods regularly consumed and with low day-to-day variation (e.g., breakfast, snacks, and hot drinks) in the previous 12 mo; intakes of other dairy products not covered by the food diary such as milk in tea, milk-based spread on bread, milk in porridge and cereals, chocolate milk, yogurt, cream in coffee and on fruits compote, butter on bread and cheese not in hot meals were recorded in the FFQ. The 60-min dietary interview, which was shortened to 45 min from 1 September, 1994, was performed to check for food overlap between the food diary and the FFQ and to collect information on food choices, food preparation practices, and portion sizes of the foods collected in the food diary. Each food intake amount (in grams per day) was computed by combining the information from the food diary, FFQ, and interview. Intakes of total energy and nutrients were calculated based on nutrient contents from the Swedish Food Database PC KOST2-93 of the Swedish National Food Administration. The reproducibility and validity of the diet measurement method showed reasonably well when ranking individuals according to their usual dietary intake [21,22]. For example, the energy-adjusted reproducibility correlation coefficients in males and females were as follows: milk, 0.82 and 0.70; cream, 0.48 and 0.42; and cheese, 0.71 and 0.71, respectively. The energy-adjusted validation correlation coefficients in males and females were as follows: milk, 0.83 and 0.84; cream, 0.47 and 0.52; and cheese, 0.47 and 0.59, respectively.

The exposure variables in this study included nonfermented milk, fermented milk (yogurt and sour milk), cheese, cream, and butter. Consistent with our previous studies [23,24], categories were created to cover a wide range of intake including more extreme intakes (i.e., nonfermented milk: <200, 200–400, 400–600, 600–800, 800–1000, >1000 g/d; fermented milk: 0, 0–100, 100–200, 200–300, >300 g/d; cheese: 0–20, 20–40, 40–60, 60–80, 80–100, >100 g/d; cream: 0–10, 10–20, 20–30, 30–40, 40–50, >50 g/d; butter: 0, 0–10, 10–20, 20–30, 30–40, 40–50, >50 g/d; for each category, the equal sign was included in the upper limit). Furthermore, we conducted analyses on a continuous scale of exposures (per 100 g/d for nonfermented milk and fermented milk; per 10 g/d for cheese, cream, and butter).

### Measurement of metabolites

Peripheral venous fasting blood samples were collected at enrollment (1991–1994) and plasma was stored at −80 °C until metabolomic analysis in 2020. A total of 1372 biochemicals (including 835 named metabolites, 268 unnamed metabolites, and 269 xenobiotics) were measured on the Metabolon Platform (Morrisville) using a well-validated untargeted liquid chromatography coupled to tandem mass spectrometry platform. Named and unnamed metabolites with >75% of values missing were excluded. Xenobiotics with missing values were imputed with 0. All metabolites were transformed using the natural log as in previous work, and those with values beyond ±5 SD away from the mean of that metabolite were recorded to the 5-SD threshold [25]. After these exclusions, 991 metabolites were available for metabolomic analysis.

### Follow-up and ascertainment of T2D

Incident diabetes was ascertained based on the Swedish Prescribed Drug Register (ATC code A10 or insulin use) and the International Classification of Diseases 10th Revision codes E10-E14 and O244-O249, as recorded in the Swedish National Diabetes Register, the Swedish Inpatient Register, the Swedish Outpatient Register, the Swedish Cause of Death Register, the Malmö HbA1c register, the regional Diabetes 2000 register of the Scania region, and the ANDIS (All New Diabetics in Scania) study. Apart from being ascertained in these registers, some incident diabetes was identified by reexamination screenings of the study participants, as described in previous work [26,27]. Participants were followed up from the baseline assessment date to the date of first diabetes onset, death, emigration, or the conclusion of the follow-up period (31 December, 2020), whichever occurred first. The rate of loss to follow-up due to emigration from Sweden was 0.76% (*n* = 222). Cases with incident type 1 diabetes (*n* = 176), latent autoimmune diabetes in adults (*n* = 15), secondary diabetes (*n* = 5), and other types of diabetes (*n* = 11) were considered non-T2D cases. In line with our previous project [27], those with incident non-T2D have been censored at the date of diagnosis.

### Assessment of covariates

Information on date of birth (to compute age) and sex was collected by a 10-digit national personal identification number. Smoking status (current, former, or never), levels of educational attainment (elementary, primary and secondary, upper secondary, further education without a degree, and university degree), a family history of diabetes, lipid-lowering medication, and a personal history of hypertension were self-reported and collected by a standardized questionnaire. Alcohol consumption, derived from the 7-d food diary and FFQ, was categorized into sex-specific quintiles after zero-consumer was taken care of. Physical activity was assessed by a questionnaire regarding leisure time physical activity [28] and allocated into <7.5, 7.5–15, 15–25, 25–50, and ≥50 metabolic equivalent task hours per week. Weight and height were measured at recruitment following standardized processes. BMI was calculated as weight (in kilograms) divided by squared height (in meters). Blood pressure was measured using a mercury sphygmomanometer. Hypertension was defined as systolic/diastolic blood pressure of ≥140/90 mm Hg and/or having a self-reported history of hypertension. A personal history of prevalent cardiovascular disease (CVD; including coronary events or stroke) and cancer was ascertained using information from registries. The dietary assessment version (method) referred to the dietary interview time change because slightly altered coding routines were introduced in September 1994 to shorten the interview time (from 60 to 45 min). However, the altered coding routines did not have a major influence on the intake ranking of individuals [29]. The season represented the season of diet data collection (spring, summer, autumn, or winter).

### Statistical analysis

Continuous variables were presented by means ± SDs or medians (IQRs, interquartile ranges) and categorical variables by percentages for descriptive purposes.

#### Analysis 1 (dairy intake and T2D)

Cox proportional hazards regression models were used to examine associations of dairy intake with risk of T2D. The hazard ratios (HRs) and corresponding 95% confidence intervals (CIs) were computed. Four models were built, and covariate selection for models was guided by a directed acyclic graph (Supplemental Figure 2) [30]. Model 1 was adjusted for age, sex, dietary assessment version (method), season, and total energy intake. Model 2 was adjusted for variables in model 1 plus leisure-time physical activity, alcohol consumption, smoking status, and educational level. Model 3 was adjusted for variables in model 2 plus a family history of diabetes, lipid-lowering medication, hypertension at baseline, a personal history of CVD, a personal history of cancer, and intake of fiber, vegetable and fruit, meat, soft drinks, and coffee. The type of energy adjustment used was the standard model, which estimated the average relative causal effect of the exposure (i.e., the effect of substituting the exposure with other calorific sources to maintain the same total energy) [31]. Model 4 was additionally adjusted for BMI. Model 3 was considered the main model in the analyses because BMI may be a potential mediator. *P* for trend was calculated by treating categories of dairy intake as ordinal variables in the model. The proportional hazard assumption was tested by including interaction terms between dairy products and log follow-up time in the multivariable models, and no violation was observed (*P* > 0.05 for all). Furthermore, restricted cubic splines with 3 knots (percentiles at 30, 60, and 90) were used to assess the dose–response association between dairy intake and risk of T2D, with reference at 0 g [32]. Adjusted survival curves with inverse probability weights were created to better understand how the risk may change over time [33,34].

Because a previous study in MDCS has shown an interaction of dairy products (cheese) with sex in relation to T2D risk [6], we performed a preplanned stratified analysis by sex. Confounding adjustment in the analysis followed model 3 except for sex, as described earlier. Interactions were assessed by adding interaction terms between dairy products and sex in Cox models.

Several sensitivity analyses were conducted. First, the percentage of total energy from carbohydrates was additionally controlled for [35]. Second, participants with prevalent CVD or cancer at baseline were excluded, as these conditions could result in important dietary changes. Third, potential misreporters of energy intake [36] or those with substantial diet changes before baseline examinations [37] were excluded. Fourth, to reduce reverse causation bias, we excluded T2D cases that developed during the first 2 years of follow-up. Fifth, to minimize potential residual confounding of socioeconomic status, we additionally adjusted for the socioeconomic classification index, which was based on information on job title, tasks, and position at work [37,38]. Sixth, to investigate whether socioeconomic status can modify the associations between dairy products and T2D, we examined interactions between the socioeconomic classification index and dairy products by using the likelihood ratio test. Finally, to quantify the potential effect of unobserved confounding, we calculated the E-values [39].

#### Analysis 2 (dairy intake and plasma metabolites)

We first standardized each metabolite to a mean of 0 and SD of 1. To normalize the distribution, dairy intake was also naturally log-transformed after adding 0.01 to zero values. To identify metabolites correlated to dairy intake, participants were randomly split into training/crossvalidation (70%) and testing sets (30%). In the training set, elastic net regression model with a 10-fold crossvalidation was used to regress dairy intake on the 991 metabolites [40]. Then, we applied the trained model to the testing set to calculate a metabolite profile related to dairy intake. The profile was calculated as the weighted sum of the identified metabolites with weights equal to the elastic net regression coefficients. In the training set, to avoid overfitting, elastic net regression model with a leave-1-out approach was used to calculate the metabolite profile. Similar methods were used in other studies creating metabolomic profiles reflecting dietary intake [10,41,42]. To investigate whether the profile is specific to the food variable, we calculated Spearman correlation coefficients (*r*) among dairy metabolite profiles and dairy products as well as other foods. Furthermore, we showed the *r* of individual metabolites identified from the elastic net regression models and dairy products as well as other foods.

Among the identified metabolites in the elastic net regression model, we further assessed the associations of metabolites with dairy intake using linear regression models. The adjusted covariates included age; sex; season; total energy intake; leisure-time physical activity; alcohol consumption; smoking status; education; a family history of diabetes; use of lipid-lowering drugs; hypertension at baseline; a history of CVD; a history of cancer; consumption of fiber, vegetable and fruit, meat, soft drinks, and coffee; BMI; and baseline diabetes. Bonferroni-corrected *P* value (0.05/the number of metabolites) was used.

All analyses were performed with SAS software, version 9.4, and R, version 4.2.3—glmnet package [40], corrplot package, and adjustedCurves package [43], at a 2-sided α = 0.05. Hazards are interpreted as risks in this study.

## Results

### Baseline characteristics

Table 1 shows the baseline characteristics of the 26,461 participants (38.7% males). The means ± SDs of age and BMI were 58.0 ± 7.6 y and 25.6 ± 3.9 kg/m^2^, respectively. Compared with participants who did not develop T2D, those with incident T2D were more likely to be males, to have a higher BMI, to have lower education, to take lipid-lowering medication, and to have hypertension, CVD, and a family history of diabetes, whereas they were less likely to have cancer. Supplemental Tables 1–5 present the baseline study population characteristics according to categories of dairy intake.

**TABLE 1:** Baseline characteristics of the study participants (*N* = 26,461)^1^.

Characteristics	Total	Incident type 2 diabetes	
		No	Yes
No. of participants	26,461	21,909	4552
Age (y)	58.0 ± 7.6	58.1 ± 7.7	57.5 ± 7.1
Sex: male (%)	38.7	37.0	46.9
BMI (kg/m^2^)	25.6 ± 3.9	25.2 ± 3.7	27.7 ± 4.2
University degree (%)	14.5	15.2	11.0
Zero-consumers of alcohol (%)	6.05	5.87	6.94
High leisure-time physical activity (%)	52.7	53.4	49.1
Smoking status (%)			
Current	28.4	28.2	29.1
Former	33.5	33.1	35.4
Never	38.1	38.7	35.5
Lipid-lowering medication (%)	2.93	2.57	4.64
Hypertension (%)	60.6	58.1	72.5
Cardiovascular disease (%)	2.77	2.61	3.56
Cancer (%)	6.14	6.27	5.56
Family history of diabetes (%)	1.86	1.71	2.61
Total energy intake (kcal/d)	2190 (1825, 2636)	2185 (1823, 2622)	2220 (1837, 2695)
Fiber (g/1000 kcal per day)	9.0 (7.4, 10.8)	9.0 (7.5, 10.8)	8.8 (7.4, 10.6)
Vegetables and fruits (g/d)	346.0 (245.4, 473.3)	347.4 (246.7, 474.4)	338.4 (240.0, 465.5)
Meat (g/d)	123.8 (90.8, 164.4)	122.0 (89.1, 161.7)	132.5 (98.6, 177.5)
Soft drinks (g/d)	8.5 (0, 94.3)	6.1 (0, 94.3)	21.4 (0, 104.3)
Coffee (g/d)	450.0 (250.0, 675.0)	450.0 (257.1, 675.0)	450.0 (228.6, 675.0)
Nonfermented milk (g/d)	230.0 (85.7, 399.7)	225.7 (83.1, 392.9)	253.2 (99.1, 430.6)
Fermented milk (g/d)	50.0 (0, 142.9)	53.6 (0, 142.9)	42.9 (0, 125.0)
Cheese (g/d)	39.4 (22.7, 60.0)	39.6 (22.9, 60.0)	38.8 (21.7, 60.0)
Cream (g/d)	10.7 (4.1, 20.5)	10.9 (4.3, 20.8)	9.4 (3.2, 18.6)
Butter (g/d)	0 (0, 14.9)	0 (0, 15.8)	0 (0, 11.5)

1 Continuous variables are expressed as means ± SDs or medians (IQRs) and categorical variables as %.

### Dairy intake and risk of T2D

**FIGURE 1:**
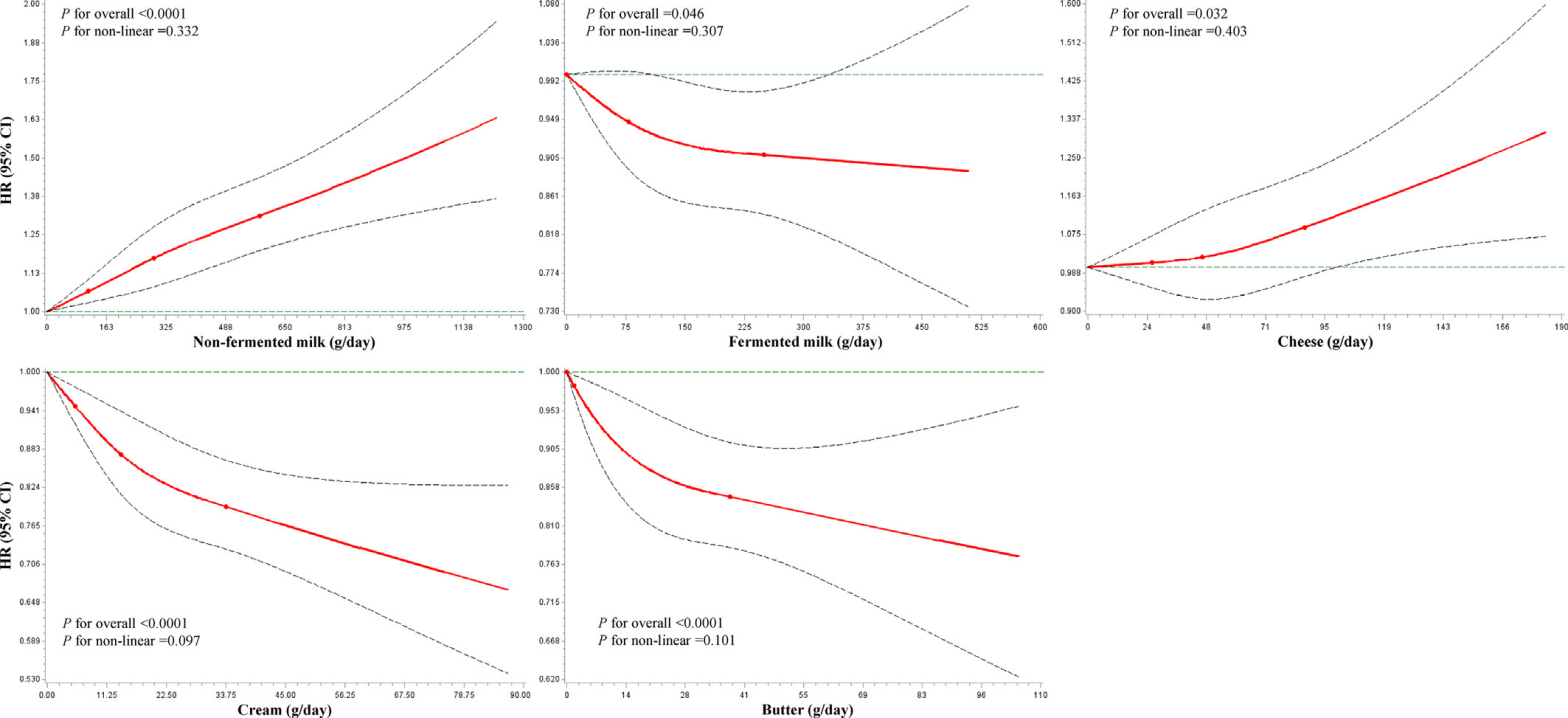
Restricted cubic spline analysis of the association between dairy intake and risk of type 2 diabetes. Estimates were calculated in Cox proportional hazards models, adjusted for age, sex, dietary assessment version (method), season, total energy intake, leisure-time physical activity, alcohol consumption, smoking status, educational level, a family history of diabetes, lipid-lowering medication, hypertension at baseline, a personal history of cardiovascular disease, a personal history of cancer, fiber, vegetable and fruit, meat, soft drinks, and coffee. Solid lines are hazard ratios, and dashed lines are 95% CIs. The knots located at the 30th, 60th, and 90th percentiles of exposure and the lowest values of dairy intake (0 g/d) were used as the reference values. The distributions of dairy intake were winsorized at the 99.5 percentiles before modeling.

During 541,550 person-years of follow-up (median: 24.3 y; IQR: 14.9, 26.6 y; maximum 29.8 y), 4552 incident T2D cases were recorded among 26,461 participants (17.2%). Table 2 lists the association between dairy intake and risk of T2D. In the main model (model 3) adjusting for demographic characteristics, lifestyle factors, and dietary intake, participants who consumed high amounts of nonfermented milk (>1000 g/d compared with <200 g/d; HR: 1.40; 95% CI: 1.12, 1.74; *P*-trend < 0.0001) and cheese (>100 g/d compared with <20 g/d; HR: 1.23; 95% CI: 1.07, 1.41; *P*-trend = 0.04) had higher T2D risk, whereas those who consumed high amounts of fermented milk (>300 g/d compared with 0 g/d; HR: 0.88; 95% CI: 0.74, 1.03; *P*-trend =0.03), cream (>50 g/d compared with <10 g/d; HR: 0.77; 95% CI: 0.64, 0.92; *P*-trend <0.0001), and butter (>50 g/d compared with 0 g/d; HR: 0.82; 95% CI: 0.71, 0.94; *P*-trend <0.0001) experienced lower T2D risk. Spline regression models showed linear associations between all investigated dairy products and risk of T2D (all *P*-overall ≤ 0.03; all *P*-nonlinearity ≥ 0.10) (Figure 1). In continuous analyses, per 100 g/d increase in nonfermented milk was associated with 4% higher risk of T2D (HR: 1.04; 95% CI: 1.03, 1.05), and fermented milk was associated with 3% lower risk of T2D (HR: 0.97; 95% CI: 0.94, 1.00). The HRs (95% CIs) per 10 g/d were 1.01 (1.00, 1.02) for cheese, 0.95 (0.93, 0.97) for cream, and 0.97 (0.95, 0.99) for butter. Such associations were attenuated after additional adjustment for BMI. Supplemental Figure 3 shows adjusted survival curves.

**TABLE 2:** Association between dairy intake and risk of type 2 diabetes (*N* = 26,461)^1^.

	Intake categories							*P*-trend	Continuous
	1	2	3	4	5	6	7		
Nonfermented milk (g/d)	0–200	200–400	400–600	600–800	800–1000	>1000	—		Per 100 g/d
No. of participants	11,789	8072	4204	1499	501	396	—		
No. of cases	1883	1382	780	304	112	91	—		
Person-years	248,045	165,431	83,154	28,573	9457	6891	—		
Incidence per 1000 person-years	7.59	8.35	9.38	10.64	11.84	13.21	—		
Model 1	1.00 (reference)	1.13 (1.05, 1.21)	1.27 (1.17, 1.39)	1.44 (1.27, 1.63)	1.61 (1.33, 1.96)	1.74 (1.41, 2.16)	—	<0.0001	1.06 (1.05, 1.07)
Model 2	1.00 (reference)	1.10 (1.02, 1.18)	1.20 (1.10, 1.30)	1.29 (1.14, 1.47)	1.44 (1.19, 1.75)	1.46 (1.18, 1.82)	—	<0.0001	1.04 (1.03, 1.06)
Model 3	1.00 (reference)	1.08 (1.01, 1.16)	1.17 (1.08, 1.28)	1.26 (1.11, 1.43)	1.45 (1.19, 1.77)	1.40 (1.12, 1.74)	—	<0.0001	1.04 (1.03, 1.05)
Model 4	1.00 (reference)	1.05 (0.98, 1.12)	1.10 (1.01, 1.20)	1.11 (0.98, 1.26)	1.25 (1.02, 1.51)	1.15 (0.93, 1.44)	—	<0.01	1.02 (1.01, 1.03)
Fermented milk (g/d)	0	0–100	100–200	200–300	>300	—	—		Per 100 g/d
No. of participants	9239	7982	5766	2405	1069	—	—		
No. of cases	1760	1340	914	377	161	—	—		
Person-years	180,320	166,221	121,657	50,710	22,641	—	—		
Incidence per 1000 person-years	9.76	8.06	7.51	7.43	7.11	—	—		
Model 1	1.00 (reference)	0.90 (0.84, 0.97)	0.83 (0.77, 0.90)	0.81 (0.72, 0.90)	0.76 (0.65, 0.90)	—	—	<0.0001	0.93 (0.90, 0.95)
Model 2	1.00 (reference)	0.96 (0.89, 1.03)	0.91 (0.84, 0.98)	0.89 (0.80, 1.00)	0.84 (0.71, 0.98)	—	—	<0.01	0.96 (0.93, 0.98)
Model 3	1.00 (reference)	0.97 (0.90, 1.04)	0.93 (0.85, 1.00)	0.94 (0.84, 1.05)	0.88 (0.74, 1.03)	—	—	0.03	0.97 (0.94, 1.00)
Model 4	1.00 (reference)	0.97 (0.90, 1.04)	0.93 (0.86, 1.01)	0.98 (0.87, 1.09)	0.90 (0.77, 1.06)	—	—	0.12	0.98 (0.95, 1.01)
Cheese (g/d)	0-20	20-40	40-60	60-80	80-100	>100	—		Per 10 g/d
No. of participants	5611	8177	6099	3231	1681	1662	—		
No. of cases	1028	1386	1037	538	265	298	—		
Person-years	107,237	166,817	126,915	68,773	36,374	35,434	—		
Incidence per 1000 person-years	9.59	8.31	8.17	7.82	7.29	8.41	—		
Model 1	1.00 (reference)	0.89 (0.82, 0.97)	0.90 (0.82, 0.98)	0.87 (0.78, 0.96)	0.81 (0.70, 0.93)	0.97 (0.84, 1.11)	—	0.06	0.99 (0.98, 1.00)
Model 2	1.00 (reference)	0.94 (0.87, 1.02)	0.97 (0.89, 1.06)	0.96 (0.86, 1.07)	0.90 (0.78, 1.03)	1.08 (0.95, 1.24)	—	0.85	1.00 (0.99, 1.01)
Model 3	1.00 (reference)	0.97 (0.89, 1.05)	1.03 (0.94, 1.12)	1.02 (0.92, 1.14)	0.95 (0.83, 1.10)	1.23 (1.07, 1.41)	—	0.04	1.01 (1.00, 1.02)
Model 4	1.00 (reference)	0.96 (0.89, 1.04)	1.01 (0.92, 1.11)	0.99 (0.89, 1.11)	0.92 (0.80, 1.06)	1.14 (0.99, 1.31)	—	0.35	1.00 (0.99, 1.01)
Cream (g/d)	0–10	10–20	20–30	30–40	40–50	>50	—		Per 10 g/d
No. of participants	12,636	6995	3434	1639	840	917	—		
No. of cases	2361	1140	549	235	133	134	—		
Person-years	254,793	144,986	71,496	34,395	17,007	18,874	—		
Incidence per 1000 person-years	9.27	7.86	7.68	6.83	7.82	7.10	—		
Model 1	1.00 (reference)	0.86 (0.80, 0.92)	0.84 (0.76, 0.92)	0.73 (0.64, 0.83)	0.82 (0.69, 0.98)	0.73 (0.61, 0.87)	—	<0.0001	0.94 (0.92, 0.96)
Model 2	1.00 (reference)	0.90 (0.84, 0.97)	0.87 (0.80, 0.96)	0.77 (0.67, 0.88)	0.85 (0.72, 1.02)	0.75 (0.63, 0.89)	—	<0.0001	0.95 (0.93, 0.97)
Model 3	1.00 (reference)	0.91 (0.85, 0.98)	0.87 (0.79, 0.96)	0.78 (0.68, 0.89)	0.87 (0.73, 1.04)	0.77 (0.64, 0.92)	—	<0.0001	0.95 (0.93, 0.97)
Model 4	1.00 (reference)	0.94 (0.88, 1.01)	0.92 (0.84, 1.01)	0.84 (0.73, 0.96)	0.93 (0.78, 1.11)	0.84 (0.70, 1.00)	—	<0.01	0.97 (0.95, 0.99)
Butter (g/d)	0	0–10	10–20	20–30	30–40	40–50	>50		Per 10 g/d
No. of participants	15,049	3687	2192	1787	1351	772	1623		
No. of cases	2794	572	353	260	204	118	251		
Person-years	306,400	78,193	45,275	36,981	27,142	15,790	31,769		
Incidence per 1000 person-years	9.12	7.32	7.80	7.03	7.52	7.47	7.90		
Model 1	1.00 (reference)	0.84 (0.77, 0.92)	0.88 (0.79, 0.99)	0.80 (0.71, 0.91)	0.84 (0.73, 0.97)	0.81 (0.67, 0.97)	0.83 (0.73, 0.95)	<0.0001	0.97 (0.95, 0.99)
Model 2	1.00 (reference)	0.90 (0.82, 0.98)	0.91 (0.82, 1.02)	0.81 (0.71, 0.92)	0.83 (0.72, 0.96)	0.78 (0.65, 0.94)	0.78 (0.68, 0.90)	<0.0001	0.96 (0.95, 0.98)
Model 3	1.00 (reference)	0.91 (0.83, 0.99)	0.94 (0.84, 1.05)	0.84 (0.74, 0.96)	0.86 (0.75, 1.00)	0.83 (0.69, 1.01)	0.82 (0.71, 0.94)	<0.0001	0.97 (0.95, 0.99)
Model 4	1.00 (reference)	0.92 (0.84, 1.01)	0.97 (0.87, 1.08)	0.87 (0.77, 0.99)	0.91 (0.78, 1.05)	0.92 (0.77, 1.11)	0.86 (0.75, 0.99)	<0.01	0.98 (0.96, 0.99)

Model 1: adjusted for age, sex, dietary assessment version (method), season, and total energy intake. Model 2: adjusted for variables in model 1 plus leisure-time physical activity, alcohol consumption, smoking status, and educational level. Model 3: adjusted for variables in model 2 plus a family history of diabetes, lipid-lowering medication, hypertension at baseline, a personal history of cardiovascular disease, a personal history of cancer, fiber, vegetable and fruit, meat, soft drinks, and coffee. Model 4: adjusted for variables in model 3 plus BMI.

1 Values are given as hazard ratios and 95% CIs within parentheses, calculated using Cox models.

There were no substantial differences observed in subgroups defined by sex for nonfermented milk, fermented milk, cream, and butter for T2D risk (Supplemental Table 6). In contrast, the positive association between cheese and T2D was observed only in males, but not in females (*P*-interaction = 0.01).

The results did not substantially alter when additionally adjusting for carbohydrates (Supplemental Table 7), excluding participants with prevalent CVD or cancer at baseline (Supplemental Table 8), excluding participants who misreported their energy intake or those who had substantial diet change (Supplemental Table 9), excluding T2D cases who developed in the first 2 y of follow-up (Supplemental Table 10), and additionally adjusting for socioeconomic classification index (Supplemental Table 11). In addition, there was no interaction between the socioeconomic classification index and dairy products to T2D (all *P*-interaction >0.31), suggesting that the dairy–T2D association was independent of socioeconomic status. The E-values for point estimates ranged from 1.09 to 1.23 (Supplemental Table 12), indicating that an unmeasured confounder would need to be associated with dairy intake and T2D by a magnitude of ≥1.09-fold, beyond the measured confounders in multivariable Cox models, to explain away any associations observed.

### Plasma metabolites related to dairy intake

**FIGURE 2:**
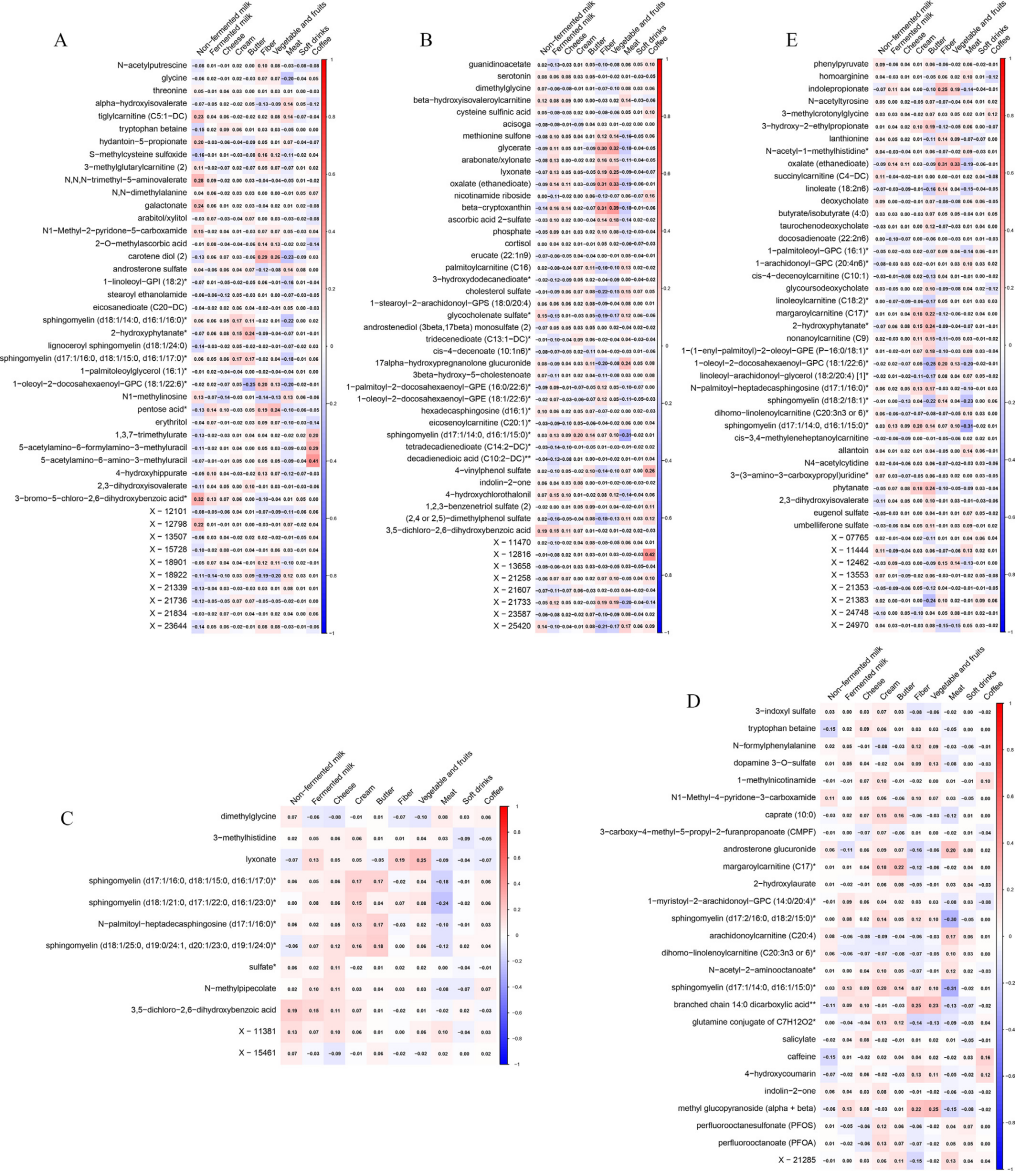
Heatmap showing Spearman correlation coefficients of individual metabolites related to dairy intake and dairy products as well as other foods: (A) nonfermented milk, (B) fermented milk, (C) cheese, (D) cream, and (E) butter.

**FIGURE 3:**
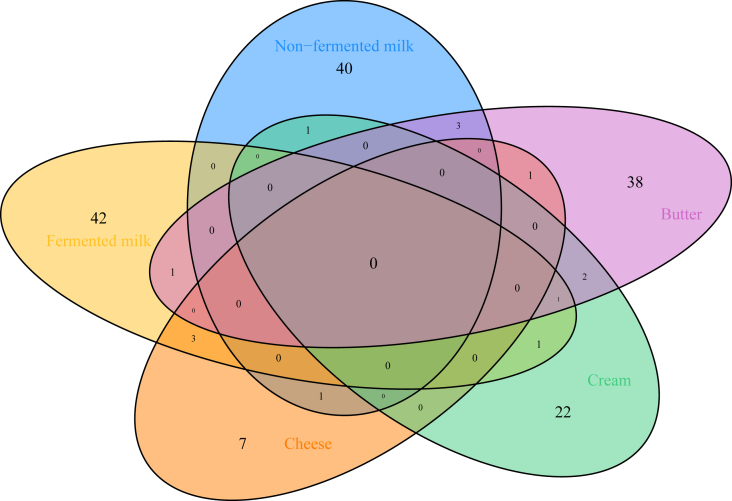
Venn diagram showing the overlapping and different metabolites for 5 types of dairy products identified using elastic net regression models.

For nonfermented milk, 45 metabolites were identified from elastic net regression (Figure 2, Supplemental Table 13). These metabolites consisted of 11 amino acids, 2 carbohydrates, 3 cofactors and vitamins, 10 lipids, 1 nucleotide, 1 partially characterized molecule, 7 xenobiotics, and 10 unnamed metabolites. The strongest positive associations were observed for 3-bromo-5-chloro-2,6-dihydroxybenzoic acid, galactonate, and N,N,N-trimethyl-5-aminovalerate, whereas the strongest inverse associations were X-21736, and tryptophan betaine, and carotene diol. For fermented milk, the identified 48 metabolites related to intake included amino acids (*n* = 7), carbohydrates (*n* = 3), cofactors and vitamins (*n* = 4), energy (*n* = 1), lipids (*n* = 19), xenobiotics (*n* = 6), and unnamed metabolites (*n* = 8) (Figure 2, Supplemental Table 14). The strongest positive associations were observed for arabonate/xylonate, oxalate (ethanedioate), and 4-hydroxychlorothalonil, and the strongest inverse associations were for X-11470, nicotinamide riboside, and glycocholenate sulfate. For cheese, 12 metabolites from 5 metabolic pathways were selected: amino acids (*n* = 2), carbohydrate (*n* = 1), lipids (*n* = 4), xenobiotics (*n* = 3), and unknown (*n* = 2) (Figure 2, Supplemental Table 15). The strongest positive associations were observed for N-methylpipecolate, 3,5-dichloro-2,6-dihydroxybenzoic acid, and N-palmitoyl-heptadecasphingosine (d17:1/16:0), and the strongest inverse associations were dimethylglycine. For cream, 27 metabolites from 6 metabolic pathways were identified: amino acids (*n* = 4), cofactors and vitamins (*n* = 2), lipids (*n* = 12), partially characterized molecule (*n* = 1), xenobiotics (*n* = 7), and unknown (*n* = 1) (Figure 2, Supplemental Table 16). The strongest positive associations were observed for sphingomyelin (d17:1/14:0, d16:1/15:0), perfluorooctanesulfonate, and androsterone glucuronide, and the strongest inverse associations were N-formylphenylalanine, salicylate, and dihomo-linolenoylcarnitine (C20:3n–3 or n–6). For butter, 46 metabolites related to cream came from 6 metabolic pathways: amino acids (*n* = 8), cofactors and vitamins (*n* = 1), energy (*n* = 1), lipids (*n* = 21), nucleotide (*n* = 3), xenobiotics (*n* = 4), and unknown (*n* = 8) (Figure 2, Supplemental Table 17). The strongest positive associations were observed for margaroylcarnitine (C17), sphingomyelin (d17:1/14:0, d16:1/15:0), and phytanate, and the strongest inverse associations were X-21383, 1-oleoyl-2-docosahexaenoyl-GPC (18:1/22:6), and lanthionine. Figure 3 presents Venn diagrams showing the number of overlapping and different metabolites for 5 types of dairy intake identified using elastic net regression models. No metabolites were associated with all 5 types of dairy intake.

The plasma metabolites selected from the elastic net regression models were further examined using the multivariable linear regression models, adjusting for lifestyle factors, other food intakes, and BMI. The results showed that the number of individual metabolites in linear regression associated with dairy intake decreased but with consistent directionality in elastic net regression (Supplemental Tables 13–17).

### Metabolite profiles as markers of dairy intake

**FIGURE 4:**
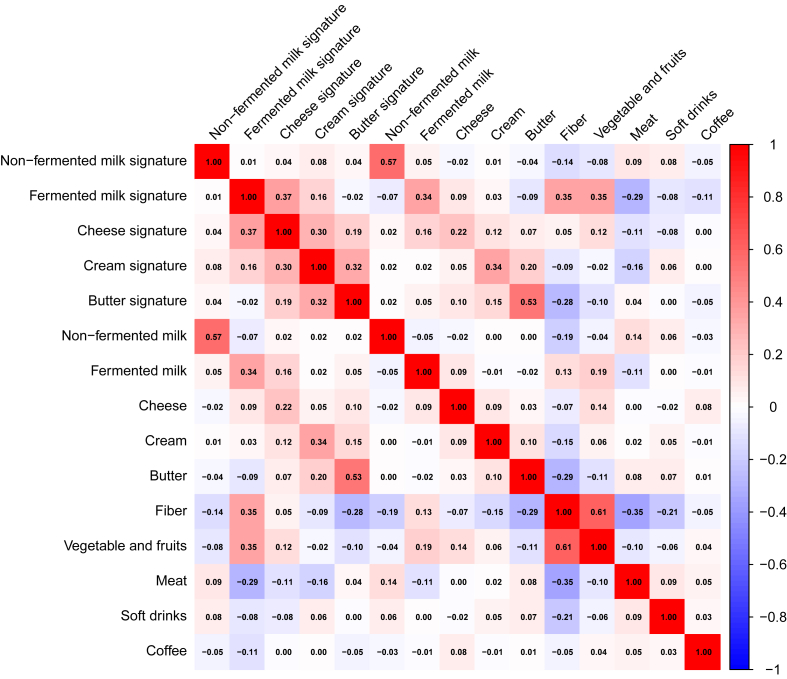
Heatmap showing Spearman correlation coefficients among dairy metabolite profile scores and dairy products as well as other foods.

Supplemental Figure 4 shows correlations between dairy products and the corresponding metabolite profiles across the training and testing sets. Overall, the metabolite profiles based on the identified individual metabolites in elastic net regression were associated with each corresponding dairy intake (Figure 4); the Spearman *r* was 0.57 for nonfermented milk, 0.34 for fermented milk, 0.22 for cheese, 0.34 for cream, and 0.53 for butter. Furthermore, the metabolite profile related to fermented milk intake was positively associated with intakes of fiber as well as vegetables and fruits (*r* = 0.35) and inversely associated with meat intake (*r* = −0.29); the butter signature was inversely associated with fiber intake (*r* = −0.28).

## Discussion

Among the 26,461 Swedish adults followed up for a median of 24.3 y, higher nonfermented milk intake and very high cheese intake (only among males) were significantly associated with higher risk of incident T2D, whereas higher intakes of fermented milk, cream, and butter were associated with a lower risk. The associations were attenuated after adjusting for BMI. In a subsample, we identified plasma metabolites for the 5 types of dairy intake and observed that different dairy products had variable metabolite profiles.

### Dairy intake and T2D

Evidence from several meta-analyses consistently documented a protective association between dairy intake (mainly yogurt) and T2D [[2], [3], [4]]. However, the studies included in these meta-analyses were heterogeneous, with different dairy intake ranges, covariate adjustments, and dietary assessment methods. For example, 2 meta-analyses suggested a positive association between milk intake and T2D risk in European populations, but an inverse association in Asian populations [3,4]. This discrepancy may be due to that Europe has higher dairy consumption than Asia. Furthermore, we observed increased risk of T2D among those above 100 g/d of cheese intake, although 0–100 g/d cheese intake was not associated. This finding is supported by previous research showing that increasing cheese consumption by >0.5 serving/d was associated with a 9% (95% CI: 2%, 16%) higher T2D risk compared with maintaining stable intakes [44]. In contrast, meta-analyses of prospective cohort studies have shown no association between cheese intake (per 10 g/d increment) and risk of T2D [3,4]. However, none of the included studies examined intakes above 100 g/d. In addition, consistent with our previous study [6], this study showed an interaction with sex and a positive association between cheese and T2D among males only. Likewise, subgroup analysis in the meta-analysis also indicated that cheese intake was associated with 5% (95% CI: 2%, 9%) higher T2D risk per 10 g/d in males [4]. Moreover, a few studies analyzed cream and butter and indicated null or inverse associations with risk of T2D [2,4]. Our results showed that high intakes of cream and butter had inverse associations with risk of T2D; the biologically plausible explanation is needed to further investigate. In addition, the dairy–fat matrix may result in unique effects of individual dairy products on T2D [45].

### Dairy intake and plasma metabolites

Most previous studies of dairy intake and metabolomics to date measured relatively small metabolite sets relative to thousands of measurable metabolites [13,14,[46], [47], [48]]. In this study, we included 991 metabolites based on untargeted plasma metabolomics data. Our results indicated that nonfermented milk, fermented milk, cheese, cream, and butter were all associated with different subclasses of sphingomyelins [positive coefficients except for sphingomyelin (d18:2/18:1) associated with butter]. Previous studies also consistently reported that sphingomyelins were associated with dairy intake [13,14,46,49,50]. These findings collectively indicate that sphingomyelins are robust biomarkers of dairy intake. Furthermore, our results showed that no metabolites identified in the study were associated with all 5 types of dairy intake, indicating that different types of dairy intake had different metabolic responses. These findings emphasize the importance of studying the associations between dairy products by type and risk of T2D.

For nonfermented milk, 3-bromo-5-chloro-2,6-dihydroxybenzoic acid and galactonate were the most positive contributors. The 3-bromo-5-chloro-2,6-dihydroxybenzoic acid is a xenobiotic and belongs to the class of organic compounds known as salicylic acids, which is a novel biomarker and warrants further investigation. Given that nonfermented milk is a lactose-containing dairy product and lactose can be digested into galactose [51], it is not surprising that galactonate has a very strong positive association with nonfermented milk. Moreover, a randomized crossover trial indicated that urinary galactose was specific for milk intake [48]. Likewise, galactose had a strong association with milk in a nested case–control study [52]. For fermented milk, most metabolites were lipids, amino acids, and some unnamed metabolites. These metabolites could be the products of either fermentation or human metabolism. In addition, arabonate/xylonate was the top metabolite related to fermented milk intake. Arabonate/xylonate is involved in the metabolic pathways of pentose metabolism, and probiotics in fermented milk may be involved in pentose metabolism. For cheese, the top metabolites N-methylpipecolate, 3,5-dichloro-2,6-dihydroxybenzoic acid, and N-palmitoyl-heptadecasphingosine (d17:1/16:0) are novel biomarkers as they have not been identified in previous studies. In addition, sphingomyelins have been previously associated with cheese intake [52], aligned with our study. Taken together, we identified many novel candidate biomarkers for different types of dairy intake, which need to be confirmed in future studies, especially in controlled feeding studies.

Studies have traditionally relied upon self-reported dairy intake that is subject to recall bias. Thus, the metabolomic scores are of potential use as biomarkers of dairy intake. Especially because dairy products contain many nutrients, combining individual metabolites into a multimetabolite profile score is more efficient than a single metabolite in predicting dairy intake. Our results indicated that dairy metabolite profile scores were associated with each corresponding dairy, indicating that these metabolite profiles may be used as objective measures of dairy intake. Interestingly, the fermented milk signature was positively associated with intakes of fiber, vegetables, and fruits but inversely associated with meat intake. This is supported by previous studies showing that fermented milk intake was associated with higher consumption of fruits, vegetables, and whole grains [53]. Nevertheless, we did not examine the associations of metabolite profile scores related to dairy intake with T2D, because our sample with metabolomics included very few new-onset incident T2D cases, which limited the statistical power to draw a firm conclusion. Thus, our work needs replication in other large-scale cohorts.

### Strengths and limitations

Strengths of our study include the large sample size with nearly 3 decades of follow-up, the large number of incident T2D cases, and the high follow-up rate (>99%). In addition, the detailed dairy data allowed us to investigate nonfermented milk, fermented milk, cheese, butter, and cream separately. Moreover, we included metabolomic data with more metabolites than any previous study to identify those related to dairy intake.

This study has several limitations. First, diet was measured once at baseline, and potential changes in dairy intake during follow-up could have occurred, which might make the observed associations with T2D null. However, diet in adulthood is relatively stable over time and measuring diet at baseline can be used to examine the effects of diet exposure on future health outcomes, especially for diseases like T2D with a long induction period [54]. Second, observational studies can be used for causal inference, but under strong assumptions [55]. In addition, the possibility of residual confounding (e.g., income and gut microbiota) cannot be ruled out. Third, metabolomic analysis was cross-sectional. Finally, the study participants were Swedish middle-aged participants, so the findings from the study may not be generalizable to populations of different ethnicities and age groups.

### Conclusions

In conclusion, among Swedish adults with generally high dairy intake, both higher nonfermented milk and cheese intakes (among men only) were associated with higher risk of incident T2D, whereas higher fermented milk, cream, and butter intakes were associated with a lower risk. These findings suggest that it is important to examine dairy products separately in relation to T2D because they differ in composition and structure. Moreover, identified metabolites provide novel insights into potential pathways underlying the associations between dairy intake and risk of T2D.

## Author contributions

The authors’ responsibilities were as follows – SZ, YB, ES: conceived and designed the study; SZ: performed the statistical analysis and wrote the manuscript; and all authors: contributed to the interpretation of the results and revision of the manuscript and read and approved the final manuscript.

## Data availability

The data that support the findings of this study are available from The Malmö Cohorts at Lund University with the permission of the MDC Steering Committee (https://www.malmo-kohorter.lu.se/malmo-cohorts). The analytic codes for main analyses are available in Supplemental Material.

## Funding

This study was funded by the Swedish Research Council (2020-01412), Heart and Lung Foundation (20190555 and 20200482), Crafoord Foundation (20210674), and Swedish Foundation for Strategic Research (IRC15-0067).

## Conflict of interest

The authors report no conflicts of interest.
