# Supplementary material for: Dairy intake, plasma metabolome, and risk of type 2 diabetes in a population-based cohort
Source: Am J Clin Nutr. 2025 Apr 3;121(5):1137–48. doi: 10.1016/j.ajcnut.2025.02.023 (PMC12107495; doi:10.1016/j.ajcnut.2025.02.023)
Supplement: Multimedia component 1 [file mmc1.docx]

**Online Supplementary Materials**

**Dairy intake, plasma metabolome, and risk of type 2 diabetes in a population-based cohort**

Authors: Shunming Zhang^*^, Suzanne Janzi, Yufeng Du, J. Gustav Smith, Lu Qi, Yan Borné^#^, Emily Sonestedt^*#^

^#^These authors are joint senior authors.

***Correspondence to:** Shunming Zhang, shunming.zhang@med.lu.se; Emily Sonestedt, emily.sonestedt@med.lu.se

**Supplementary**
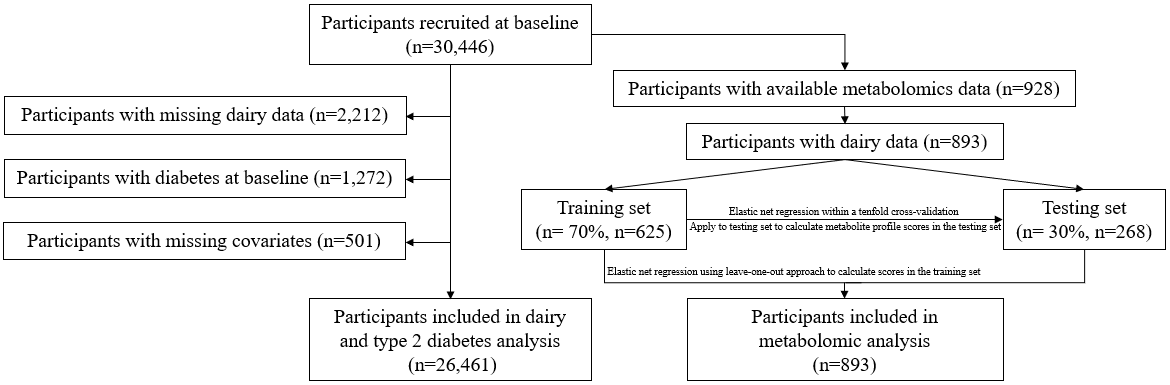
**Figure 1.** Schematic of the study design.

**Supplementary Figure 2.** Directed acyclic graph (DAG) derived from expert knowledge and literature, using DAGitty v3.1 (available at https://dagitty.net/dags.html). Each node represents a specific variable and the arrows indicate causal associations between them. The exposure variable is dairy intake and the outcome variable is type 2 diabetes. Abbreviation: SES, socioeconomic status.


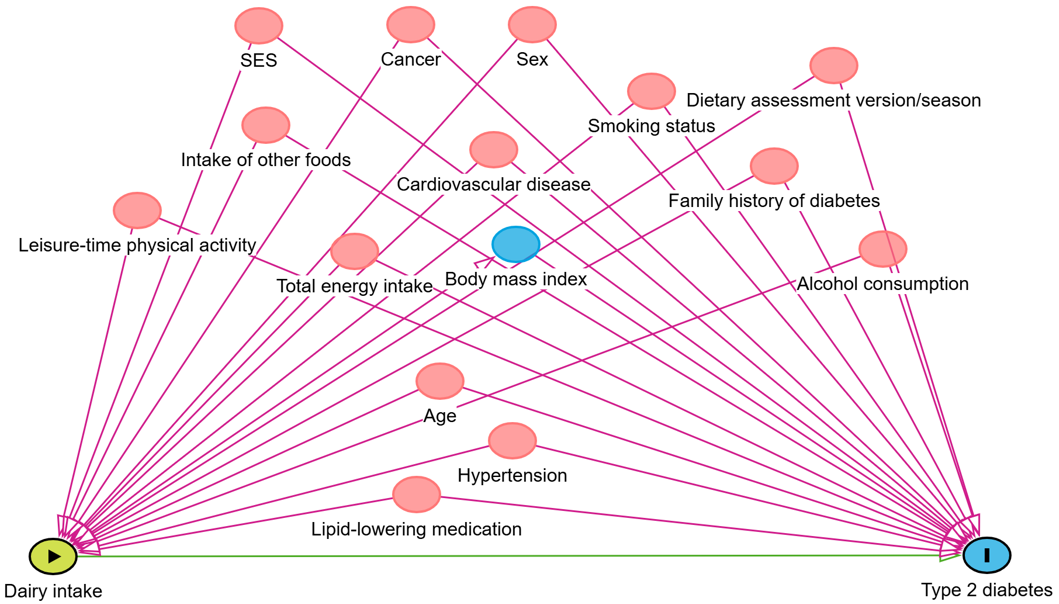


**Supplementary Figure 3.** The inverse probability weighted survival curves for the associations between dairy products and risk of type 2 diabetes, adjusting for age, sex, dietary assessment version (method), season, total energy intake, leisure-time physical activity, alcohol consumption, smoking status, educational level, family history of diabetes, lipid-lowering medication, hypertension at baseline, personal history of cardiovascular disease, personal history of cancer, fiber, vegetable and fruit, meat, soft drinks, and coffee. Notes: groups of non-fermented milk: <200, 200-400, 400-600, 600-800, 800-1000, >1000 g/day; groups of fermented milk: 0, 0-100, 100-200, 200-300, >300 g/day; groups of cheese: 0-20, 20-40, 40-60, 60-80, 80-100, >100 g/day; groups of cream: 0-10, 10-20, 20-30, 30-40, 40-50, >50 g/day; groups of butter: 0, 0-10, 10-20, 20-30, 30-40, 40-50, >50 g/day.


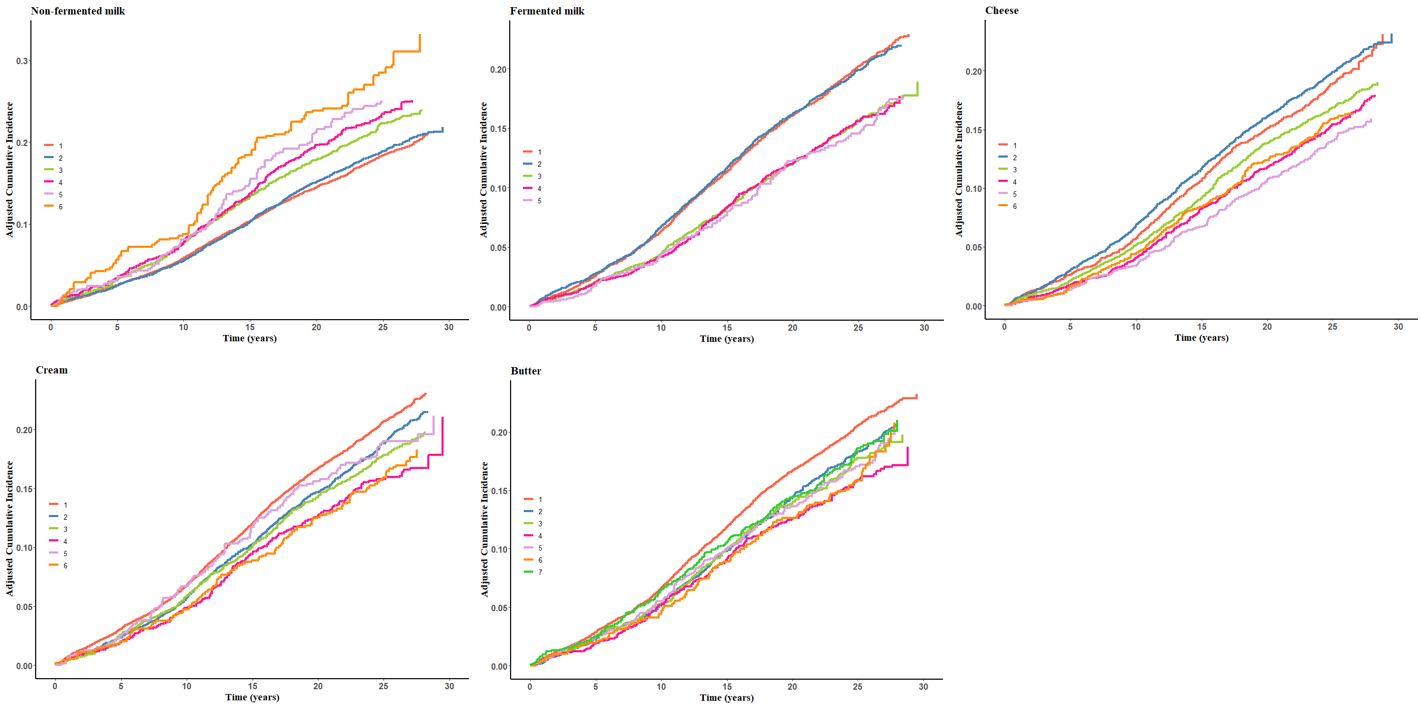


**Supplementary Figure 4.** Correlation between dairy intake and the corresponding metabolite profile score (n=893).


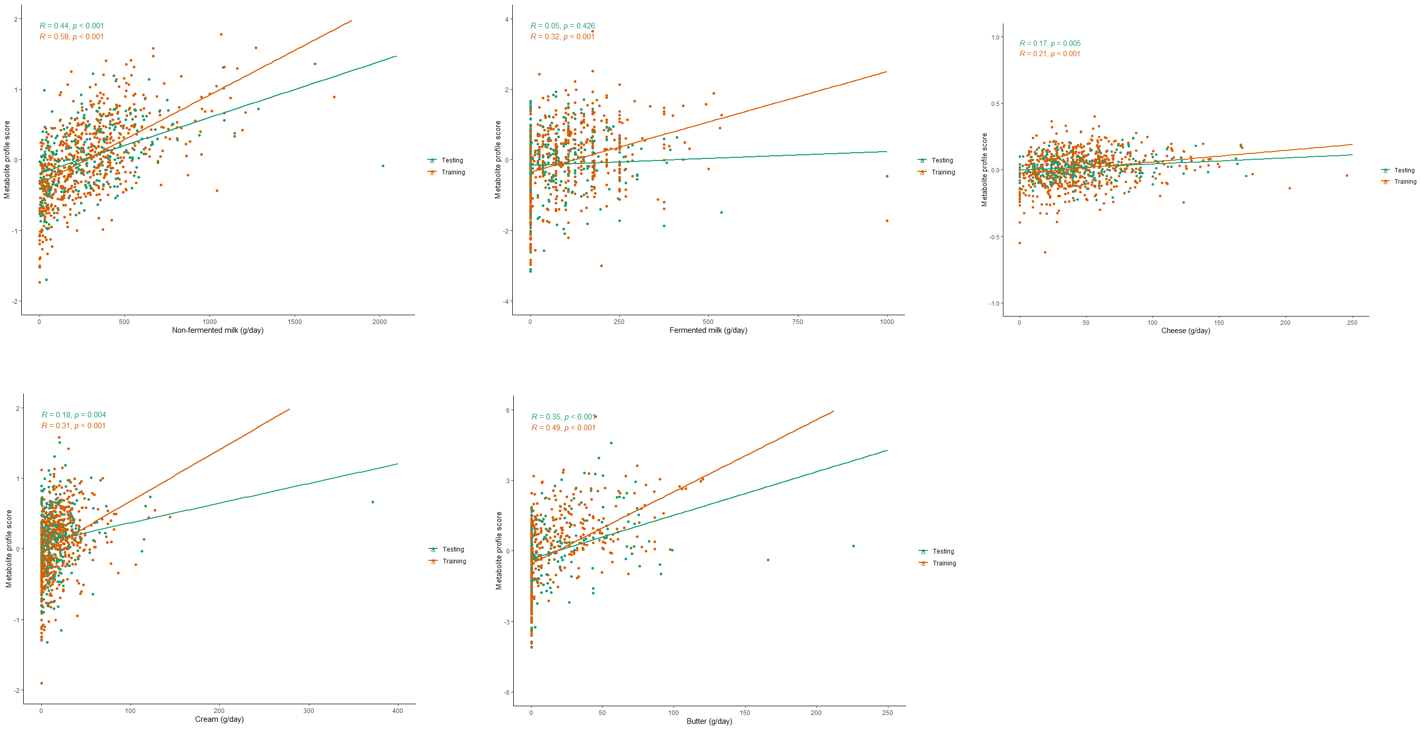


| **Supplementary Table 1.** Baseline characteristics of the study participants by categories of non-fermented milk intake (n=26,461) ^1^ | | | | | | |
| --- | --- | --- | --- | --- | --- | --- |
| Characteristics | Non-fermented milk (g/day) | | | | | |
|  | 0-200 | 200-400 | 400-600 | 600-800 | 800-1000 | >1000 |
| Number of participants | 11,789 | 8,072 | 4,204 | 1,499 | 501 | 396 |
| Age (years) | 57.3±7.5 | 58.5±7.7 | 58.7±7.7 | 58.5±7.7 | 57.7±7.6 | 57.2±6.9 |
| Sex (male, %) | 36.3 | 36.1 | 40.8 | 50.4 | 56.7 | 75.5 |
| Body mass index (kg/m^2^) | 25.4±3.8 | 25.7±3.9 | 25.9±4.0 | 26.1±4.2 | 26.5±4.3 | 26.6±4.2 |
| University degree (%) | 16.2 | 13.8 | 12.3 | 11.3 | 14.6 | 10.9 |
| Zero-consumers of alcohol (%) | 4.30 | 5.95 | 8.78 | 9.07 | 11.8 | 12.9 |
| High leisure-time physical activity (%) | 51.8 | 53.0 | 53.9 | 55.6 | 51.9 | 50.0 |
| Smoking status (%) |  |  |  |  |  |  |
| Current | 26.5 | 27.6 | 29.9 | 34.0 | 35.7 | 51.8 |
| Former | 35.9 | 32.0 | 31.3 | 30.9 | 33.1 | 27.8 |
| Never | 37.6 | 40.4 | 38.8 | 35.1 | 31.1 | 20.5 |
| Lipid-lowering medication (%) | 2.86 | 2.97 | 2.93 | 3.74 | 1.80 | 2.27 |
| Hypertension (%) | 58.8 | 61.3 | 63.0 | 63.8 | 59.9 | 62.6 |
| Cardiovascular disease (%) | 2.73 | 2.45 | 3.26 | 2.87 | 2.99 | 4.55 |
| Cancer (%) | 6.08 | 6.26 | 6.28 | 5.54 | 7.19 | 5.30 |
| Family history of diabetes (%) | 1.65 | 2.03 | 1.97 | 2.13 | 2.00 | 2.27 |
| Total energy intake (kcal/day) | 2063 (1725, 2469) | 2193 (1847, 2602) | 2329 (1967, 2777) | 2576 (2145, 3053) | 2775 (2263, 3233) | 3116 (2578, 3655) |
| Fiber (g/1000 kcal per day) | 9.3 (7.7, 11.3) | 9.0 (7.5, 10.7) | 8.7 (7.2, 10.3) | 8.2 (6.7, 9.9) | 7.9 (6.5, 9.4) | 6.7 (5.4, 8.5) |
| Vegetables and fruits (g/day) | 349.7 (246.6, 482.2) | 344.0 (250.0, 464.2) | 350.9 (245.7, 478.2) | 336.9 (235.1, 465.9) | 324.4 (220.4, 448.2) | 277.1 (194.6, 404.0) |
| Meat (g/day) | 120.9 (87.2, 160.3) | 122.0 (91.2, 160.8) | 126.3 (94.3, 168.0) | 137.8 (99.4, 180.6) | 140.2 (99.4, 186.9) | 160.0 (119.4, 214.7) |
| Soft drinks (g/day) | 1.4 (0, 85.7) | 15.0 (0, 94.3) | 28.6 (0, 114.3) | 21.4 (0, 128.6) | 21.4 (0, 137.1) | 7.1 (0, 118.6) |
| Coffee (g/day) | 450.0 (250.0, 675.0) | 450.0 (285.7, 675.0) | 450.0 (285.7, 675.0) | 450.0 (300.0, 700.0) | 450.0 (300.0, 771.4) | 500.0 (219.6, 928.6) |
| Non-fermented milk (g/day) | 71.3 (27.8, 133.8) | 290.4 (242.9, 341.6) | 479.6 (436.4, 529.8) | 670.8 (630.7, 724.9) | 877.8 (832.9, 934.5) | 1139.6 (1059.0, 1286.3) |
| Fermented milk (g/day) | 64.3 (0, 167.9) | 53.6 (0, 139.3) | 42.9 (0, 121.4) | 35.7 (0, 107.1) | 32.0 (0, 100.0) | 35.7 (0, 125.0) |
| Cheese (g/day) | 40.0 (23.3, 62.2) | 38.6 (22.9, 58.1) | 37.6 (21.7, 58) | 38.0 (20.8, 59.7) | 38.6 (20.8, 64.3) | 38.8 (20.0, 63.1) |
| Cream (g/day) | 10.8 (4.2, 20.6) | 10.8 (4.4, 21) | 10.1 (3.9, 19.7) | 10.1 (3.5, 21.2) | 10.1 (3.2, 19.5) | 9.0 (1.7, 18.2) |
| Butter (g/day) | 0 (0, 13.7) | 0 (0, 14.5) | 0 (0, 15.3) | 0 (0, 21.7) | 0 (0, 24.0) | 0 (0, 27.9) |
| ^1^ Continuous variables were expressed as means ± standard deviations or medians (interquartile ranges) and categorial variables as %. | | | | | | |

| **Supplementary Table 2.** Baseline characteristics of the study participants by categories of fermented milk intake (n=26,461) ^1^ | | | | | |
| --- | --- | --- | --- | --- | --- |
| Characteristics | Fermented milk (g/day) | | | | |
|  | 0 | 0-100 | 100-200 | 200-300 | >300 |
| Number of participants | 9,239 | 7,982 | 5,766 | 2,405 | 1,069 |
| Age (years) | 58.5±7.6 | 57.4±7.7 | 58.0±7.7 | 58.0±7.5 | 57.4±7.6 |
| Sex (male, %) | 49.4 | 30.5 | 32.5 | 37.7 | 44.3 |
| Body mass index (kg/m^2^) | 25.8±4 | 25.6±4.0 | 25.5±3.8 | 25.2±3.5 | 25.2±3.7 |
| University degree (%) | 10.1 | 14.7 | 17.0 | 20.1 | 24.6 |
| Zero-consumers of alcohol (%) | 7.37 | 5.44 | 4.98 | 5.20 | 7.0 |
| High leisure-time physical activity (%) | 49.1 | 52.8 | 55.3 | 57.6 | 58.0 |
| Smoking status (%) |  |  |  |  |  |
| Current | 33.5 | 27.4 | 24.1 | 23.9 | 24.4 |
| Former | 33.2 | 32.3 | 33.4 | 37.5 | 37.2 |
| Never | 33.3 | 40.3 | 42.5 | 38.6 | 38.4 |
| Lipid-lowering medication (%) | 3.44 | 2.79 | 2.50 | 2.37 | 2.99 |
| Hypertension (%) | 64.0 | 59.3 | 59.6 | 55.9 | 56.7 |
| Cardiovascular disease (%) | 3.56 | 2.10 | 2.24 | 2.99 | 3.27 |
| Cancer (%) | 5.86 | 6.15 | 6.38 | 6.69 | 6.08 |
| Family history of diabetes (%) | 1.48 | 2.08 | 1.96 | 2.04 | 2.62 |
| Total energy intake (kcal/day) | 2189 (1806, 2664) | 2138 (1793, 2552) | 2186 (1838, 2623) | 2268 (1905, 2704) | 2449 (2069, 2945) |
| Fiber (g/1000 kcal per day) | 8.5 (7.0, 10.3) | 9.0 (7.6, 10.9) | 9.3 (7.8, 11.1) | 9.4 (7.9, 11.3) | 9.3 (7.7, 11.3) |
| Vegetables and fruits (g/day) | 308.9 (210.6, 428.6) | 351.4 (255.5, 476.5) | 370.2 (271.1, 496.9) | 386.1 (283.7, 520.3) | 407.8 (285.6, 567) |
| Meat (g/day) | 133.6 (98.5, 178.2) | 120.8 (88.9, 159.2) | 117.6 (86.6, 155.6) | 116.0 (84.2, 156.2) | 119.1 (83.9, 158.0) |
| Soft drinks (g/day) | 7.1 (0, 99.2) | 21.4 (0, 100.0) | 6.1 (0, 85.7) | 1.4 (0, 85.7) | 4.3 (0, 85.7) |
| Coffee (g/day) | 450.0 (271.4, 700.0) | 450.0 (250.0, 675.0) | 450.0 (285.7, 675.0) | 450.0 (250.0, 675.0) | 450.0 (225.0, 675.0) |
| Non-fermented milk (g/day) | 244.7 (82.5, 429.3) | 246.6 (108.4, 404.7) | 209.3 (78.3, 369.1) | 191.8 (64.0, 350.9) | 182.6 (61.7, 360.4) |
| Fermented milk (g/day) | 0 (0, 0) | 50.0 (35.7, 71.4) | 150.0 (125.0, 175.0) | 250.0 (221.4, 250.0) | 375.0 (353.6, 446.4) |
| Cheese (g/day) | 35.8 (20.0, 56.4) | 39.7 (23.6, 60.0) | 40.7 (25.1, 62.1) | 42.6 (24.7, 63.5) | 45.1 (25.7, 73.0) |
| Cream (g/day) | 10.0 (3.5, 20.2) | 10.8 (4.5, 20.4) | 11.5 (4.7, 21.0) | 11.2 (4.3, 21.1) | 9.2 (3.0, 19.1) |
| Butter (g/day) | 0 (0, 17.9) | 0 (0, 14.3) | 0 (0, 13.5) | 0 (0, 13.2) | 0 (0, 13.3) |
| ^1^ Continuous variables were expressed as means ± standard deviations or medians (interquartile ranges) and categorial variables as %. | | | | | |

| **Supplementary Table 3.** Baseline characteristics of the study participants by categories of cheese intake (n=26,461) ^1^ | | | | | | |
| --- | --- | --- | --- | --- | --- | --- |
| Characteristics | Cheese (g/day) | | | | | |
|  | 0-20 | 20-40 | 40-60 | 60-80 | 80-100 | >100 |
| Number of participants | 5,611 | 8,177 | 6,099 | 3,231 | 1,681 | 1,662 |
| Age (years) | 59.8±7.6 | 58.7±7.7 | 57.4±7.5 | 56.4±7.3 | 56.1±7.3 | 55.5±6.9 |
| Sex (male, %) | 40.12 | 38.1 | 37.6 | 38.8 | 40.9 | 39.0 |
| Body mass index (kg/m^2^) | 25.9±4.0 | 25.7±3.9 | 25.5±3.9 | 25.4±3.8 | 25.2±3.9 | 25.2±4.0 |
| University degree (%) | 8.27 | 12.4 | 15.5 | 18.4 | 22.7 | 25.9 |
| Zero-consumers of alcohol (%) | 9.62 | 5.54 | 5.07 | 5.04 | 3.93 | 4.27 |
| High leisure-time physical activity (%) | 49.2 | 52.4 | 54.0 | 54.7 | 52.5 | 57.2 |
| Smoking status (%) |  |  |  |  |  |  |
| Current | 30.1 | 27.4 | 27.6 | 27.9 | 28.9 | 30.0 |
| Former | 31.1 | 33.3 | 33.9 | 34.6 | 35.2 | 37.7 |
| Never | 38.8 | 39.3 | 38.4 | 37.5 | 35.9 | 32.4 |
| Lipid-lowering medication (%) | 4.67 | 3.12 | 2.28 | 2.10 | 1.78 | 1.20 |
| Hypertension (%) | 67.3 | 62.7 | 57.7 | 55.9 | 57.0 | 51.0 |
| Cardiovascular disease (%) | 4.37 | 2.94 | 2.25 | 2.17 | 1.07 | 1.38 |
| Cancer (%) | 7.34 | 6.41 | 5.51 | 5.51 | 4.88 | 5.66 |
| Family history of diabetes (%) | 1.48 | 1.90 | 1.97 | 2.41 | 1.96 | 1.44 |
| Total energy intake (kcal/day) | 1954 (1618, 2357) | 2099 (1771, 2502) | 2239 (1904, 2652) | 2387 (2014, 2799) | 2520 (2129, 2993) | 2666 (2238, 3232) |
| Fiber (g/1000 kcal per day) | 9.2 (7.5, 11.3) | 9.1 (7.6, 10.9) | 8.9 (7.5, 10.6) | 8.8 (7.3, 10.4) | 8.6 (7.2, 10.4) | 8.6 (6.9, 10.6) |
| Vegetables and fruits (g/day) | 322.1 (223.7, 446.6) | 340.8 (243.4, 461.9) | 354.8 (254.0, 474.5) | 361.0 (259.2, 489.3) | 365.8 (255.9, 500.1) | 394.2 (269.3, 562.8) |
| Meat (g/day) | 122.1 (90.1, 164.2) | 124.5 (93.3, 162.9) | 125.1 (92.9, 165.9) | 124 (89.6, 166.0) | 123.9 (86.7, 164.8) | 119.8 (79.4, 163.8) |
| Soft drinks (g/day) | 5.7 (0, 100.0) | 15.4 (0, 94.3) | 10.7 (0, 94.3) | 12.2 (0, 94.3) | 6.4 (0, 85.7) | 0 (0, 74.0) |
| Coffee (g/day) | 400.0 (225.0, 600.0) | 450.0 (250.0, 675.0) | 450.0 (285.7, 675.0) | 450.0 (300.0, 750.0) | 450.0 (300.0, 750.0) | 500.0 (300.0, 800.0) |
| Non-fermented milk (g/day) | 246.1 (89.4, 421.7) | 234.4 (92.5, 397.1) | 231.3 (88.6, 394.4) | 209.1 (76.3, 378.6) | 215.5 (77.3, 392.0) | 195.6 (60.0, 400.9) |
| Fermented milk (g/day) | 28.6 (0, 125.0) | 50.0 (0, 142.9) | 53.6 (0, 146.4) | 71.4 (0, 150.8) | 68.8 (0, 160.7) | 71.4 (0, 175.0) |
| Cheese (g/day) | 12.0 (6.4, 16.9) | 30.0 (25.1, 35.2) | 48.9 (44.3, 54.1) | 68.6 (64.0, 73.9) | 88.5 (83.7, 93.8) | 122.3 (109.0, 145.5) |
| Cream (g/day) | 8.9 (3.0, 18.8) | 10.6 (4.3, 20.4) | 11.5 (4.5, 21.3) | 11.5 (4.7, 20.9) | 11.4 (4.6, 22.3) | 10.5 (4.1, 20.9) |
| Butter (g/day) | 0 (0, 13.3) | 0 (0, 13.3) | 0 (0, 15.7) | 0 (0, 17.9) | 0 (0, 23.3) | 0 (0, 18.1) |
| ^1^ Continuous variables were expressed as means ± standard deviations or medians (interquartile ranges) and categorial variables as %. | | | | | | |

| **Supplementary Table 4.** Baseline characteristics of the study participants by categories of cream intake (n=26,461) ^1^ | | | | | | |
| --- | --- | --- | --- | --- | --- | --- |
| Characteristics | Cream (g/day) | | | | | |
|  | 0-10 | 10-20 | 20-30 | 30-40 | 40-50 | >50 |
| Number of participants | 12,636 | 6,995 | 3,434 | 1,639 | 840 | 917 |
| Age (years) | 57.6±7.5 | 58.0±7.8 | 58.5±7.9 | 58.7±7.5 | 59.0±7.5 | 59.3±7.2 |
| Sex (male, %) | 38.2 | 36.4 | 38.5 | 42.3 | 47.4 | 49.8 |
| Body mass index (kg/m^2^) | 25.9±4.0 | 25.5±3.8 | 25.4±3.8 | 25.2±3.5 | 25.3±3.7 | 25.0±3.6 |
| University degree (%) | 13.7 | 15.4 | 15.7 | 15.5 | 13.8 | 12.5 |
| Zero-consumers of alcohol (%) | 7.62 | 4.57 | 4.34 | 4.94 | 5.00 | 5.13 |
| High leisure-time physical activity (%) | 51.81 | 52.8 | 53.5 | 54.4 | 56.3 | 54.1 |
| Smoking status (%) |  |  |  |  |  |  |
| Current | 30.2 | 26.0 | 26.8 | 27.0 | 28.1 | 29.7 |
| Former | 33.0 | 34.2 | 34.3 | 33.0 | 33.9 | 33.4 |
| Never | 36.8 | 39.8 | 39.0 | 40.0 | 38.0 | 37.0 |
| Lipid-lowering medication (%) | 3.35 | 2.59 | 2.91 | 2.32 | 1.67 | 1.96 |
| Hypertension (%) | 61.2 | 59.5 | 61.1 | 59.9 | 60.1 | 60.9 |
| Cardiovascular disease (%) | 3.10 | 2.57 | 2.42 | 2.26 | 2.62 | 2.07 |
| Cancer (%) | 5.82 | 6.25 | 6.90 | 6.47 | 6.19 | 6.32 |
| Family history of diabetes (%) | 1.79 | 1.80 | 1.81 | 1.89 | 3.10 | 2.40 |
| Total energy intake (kcal/day) | 2077 (1727, 2523) | 2191 (1853, 2612) | 2287 (1943, 2708) | 2427 (2076, 2863) | 2498 (2131, 2989) | 2608 (2203, 3086) |
| Fiber (g/1000 kcal per day) | 9.3 (7.6, 11.3) | 9.0 (7.5, 10.7) | 8.7 (7.4, 10.4) | 8.4 (7.1, 10.0) | 8.4 (7.1, 10.0) | 8 (6.7, 9.6) |
| Vegetables and fruits (g/day) | 338.5 (237.4, 472) | 349.3 (249.9, 468.2) | 355.0 (256.5, 481.5) | 355.8 (256.6, 470.8) | 360.9 (266.4, 496.1) | 351.1 (254.6, 478.0) |
| Meat (g/day) | 120.8 (88.2, 160.5) | 123.1 (91.3, 162.7) | 126.7 (92.9, 167.3) | 130.3 (98.5, 175.3) | 134.6 (95.5, 179.4) | 137.9 (99.5, 181.7) |
| Soft drinks (g/day) | 2.9 (0, 94.3) | 14.3 (0, 94.3) | 14.3 (0, 94.3) | 28.6 (0, 100.0) | 28.6 (0, 114.3) | 28.6 (0, 114.3) |
| Coffee (g/day) | 450.0 (250.0, 700.0) | 450 (257.1, 675.0) | 450.0 (271.4, 675.0) | 450.0 (285.7, 650.0) | 450.0 (250.0, 675.0) | 500.0 (300.0, 750.0) |
| Non-fermented milk (g/day) | 234.4 (83.1, 408.2) | 223.4 (88.4, 389.0) | 223.2 (79.6, 387.1) | 235.4 (92.9, 380.3) | 233.5 (85.5, 385.3) | 247.2 (96.3, 425.7) |
| Fermented milk (g/day) | 50.0 (0, 142.9) | 57.1 (0, 146.4) | 57.1 (0, 150.0) | 53.6 (0, 146.4) | 63.4 (0, 160.7) | 42.9 (0, 128.6) |
| Cheese (g/day) | 37.5 (21.1, 59.3) | 40 (23.8, 60.7) | 40.0 (24.9, 61.1) | 41.5 (24.8, 62.5) | 40.1 (24.1, 61.8) | 40.4 (23.3, 61.3) |
| Cream (g/day) | 3.8 (0.7, 6.7) | 14.3 (12.0, 16.8) | 24.2 (22.0, 26.8) | 34.1 (31.9, 36.8) | 44.1 (42.2, 46.7) | 61.7 (54.8, 76.8) |
| Butter (g/day) | 0 (0, 12.3) | 0 (0, 15.4) | 0 (0, 17.1) | 0.5 (0, 21.9) | 1.2 (0, 25.2) | 1.9 (0, 27.5) |
| ^1^ Continuous variables were expressed as means ± standard deviations or medians (interquartile ranges) and categorial variables as %. | | | | | | |

| **Supplementary Table 5.** Baseline characteristics of the study participants by categories of butter intake (n=26,461) ^1^ | | | | | | | |
| --- | --- | --- | --- | --- | --- | --- | --- |
| Characteristics | Butter (g/day) | | | | | | |
|  | 0.0 | 0-10 | 10-20 | 20-30 | 30-40 | 40-50 | >50 |
| Number of participants | 15,049 | 3,687 | 2,192 | 1,787 | 1,351 | 772 | 1,623 |
| Age (years) | 58.2±7.6 | 57.2±7.7 | 57.3±7.8 | 57.7±7.7 | 58.2±7.9 | 58.5±7.4 | 58.4±7.4 |
| Sex (male, %) | 38.8 | 29.3 | 33.6 | 34.9 | 41.5 | 49.09 | 63.46 |
| Body mass index (kg/m^2^) | 25.9±3.9 | 25.6±3.8 | 25.3±3.9 | 25.2±3.9 | 25.1±4.0 | 24.7±3.6 | 24.9±3.8 |
| University degree (%) | 12.7 | 20.0 | 18.1 | 17.4 | 14.4 | 12.2 | 11.5 |
| Zero-consumers of alcohol (%) | 6.64 | 4.37 | 6.02 | 5.54 | 5.63 | 4.53 | 6.10 |
| High leisure-time physical activity (%) | 52.8 | 53.0 | 51.4 | 52.1 | 54.11 | 52.2 | 52.5 |
| Smoking status (%) |  |  |  |  |  |  |  |
| Current | 25.5 | 24.7 | 29.9 | 34.1 | 36.2 | 38.5 | 43.6 |
| Former | 35.2 | 33.6 | 30.7 | 29.5 | 30.6 | 31.2 | 29.5 |
| Never | 39.3 | 41.8 | 39.5 | 36.4 | 33.2 | 30.3 | 27.0 |
| Lipid-lowering medication (%) | 4.05 | 2.06 | 1.41 | 1.57 | 0.81 | 0.91 | 0.74 |
| Hypertension (%) | 62.7 | 57.6 | 56.4 | 56.2 | 59.2 | 56.4 | 61.2 |
| Cardiovascular disease (%) | 3.27 | 2.22 | 2.19 | 1.90 | 1.78 | 2.20 | 2.22 |
| Cancer (%) | 6.39 | 5.97 | 6.52 | 5.88 | 4.44 | 5.96 | 5.55 |
| Family history of diabetes (%) | 2.03 | 2.12 | 1.37 | 1.57 | 0.96 | 1.42 | 1.73 |
| Total energy intake (kcal/day) | 2118 (1770, 2542) | 2095 (1757, 2494) | 2129 (1817, 2529) | 2221 (1901, 2565) | 2397 (2025, 2811) | 2603 (2214, 3012) | 2979 (2540, 3471) |
| Fiber (g/1000 kcal per day) | 9.4 (7.8, 11.3) | 9.3 (7.8, 11.1) | 8.8 (7.5, 10.5) | 8.4 (7.1, 9.9) | 8.0 (6.7, 9.5) | 7.6 (6.4, 8.9) | 7.0 (5.9, 8.4) |
| Vegetables and fruits (g/day) | 354.9 (253.3, 483.3) | 362.5 (260.5, 489.6) | 351.4 (246.2, 475.3) | 327.2 (237.0, 451.4) | 309.2 (220.9, 432.3) | 310.0 (211.0, 442.7) | 285.8 (190.9, 407.2) |
| Meat (g/day) | 122.5 (90.7, 162.7) | 118.3 (86.8, 156.3) | 120.9 (86.2, 159.0) | 121.2 (88.0, 159.5) | 128.4 (91.2, 169.2) | 137.3 (101.4, 178.6) | 149.6 (110.6, 205.7) |
| Soft drinks (g/day) | 7.6 (0, 94.3) | 2.7 (0, 85.7) | 14.3 (0, 85.7) | 12.2 (0, 94.3) | 21.4 (0, 114.3) | 16.7 (0, 95.0) | 21.4 (0, 140.0) |
| Coffee (g/day) | 450 (257.1, 675.0) | 450.0 (225.0, 675.0) | 428.6 (250.0, 675.0) | 450.0 (250.0, 675.0) | 450.0 (250.0, 675.0) | 450.0 (287.5, 675.0) | 450.0 (300.0, 750.0) |
| Non-fermented milk (g/day) | 234.4 (87.8, 403.1) | 198.6 (69.4, 358.9) | 219.7 (78.8, 366.8) | 223.2 (84.4, 398.9) | 248.1 (91.6, 417.3) | 253.9 (93.8, 426.6) | 277.1 (101.7, 467.5) |
| Fermented milk (g/day) | 50.0 (0, 142.9) | 71.4 (0, 150.0) | 57.1 (0, 150.0) | 50.0 (0, 125.0) | 42.9 (0, 128.6) | 46.4 (0, 139.3) | 25.0 (0, 121.4) |
| Cheese (g/day) | 38.3 (21.8, 58.9) | 39.7 (24.3, 60.1) | 37.9 (22.5, 58.0) | 38.9 (22.9, 58.2) | 40.7 (22.9, 63.5) | 43.4 (26.4, 67.5) | 45.7 (27.3, 72.9) |
| Cream (g/day) | 9.7 (3.5, 19.0) | 11.5 (5.0, 21.5) | 11.1 (4.5, 21.2) | 12 (5.5, 22.1) | 12.1 (4.9, 22.7) | 13.2 (5.0, 24.9) | 13.1 (5.6, 25.8) |
| Butter (g/day) | 0 (0, 0) | 3.8 (1.7, 6.7) | 14.8 (13.1, 17.6) | 25.3 (23.0, 27.5) | 35.0 (32.6, 37.1) | 44.7 (42.8, 48.0) | 66.2 (57.3, 80.3) |
| ^1^ Continuous variables were expressed as means ± standard deviations or medians (interquartile ranges) and categorial variables as %. | | | | | | | |

| **Supplementary Table 6.** Association between dairy intake and risk of type 2 diabetes, stratified by pre-selected sex (n=26,461) ^1^ | | | | | | | | | | |
| --- | --- | --- | --- | --- | --- | --- | --- | --- | --- | --- |
|  | Intake categories | | | | | | | *P* for trend | Continuous | *P* for interaction |
|  | 1 | 2 | 3 | 4 | 5 | 6 | 7 |  |  |  |
| Non-fermented milk (g/day) | 0-200 | 200-400 | 400-600 | 600-800 | 800-1000 | >1000 |  |  | Per 100 g/day |  |
| Male | 1.00 (reference) | 0.97 (0.87, 1.08) | 1.10 (0.97, 1.25) | 1.11 (0.93, 1.32) | 1.41 (1.10, 1.81) | 1.61 (1.27, 2.05) |  | <0.0001 | 1.04 (1.02, 1.05) | 0.13 |
| Female | 1.00 (reference) | 1.19 (1.08, 1.30) | 1.25 (1.10, 1.40) | 1.47 (1.22, 1.76) | 1.49 (1.08, 2.04) | 0.52 (0.25, 1.10) |  | <0.0001 | 1.05 (1.03, 1.07) |  |
| Fermented milk (g/day) | 0 | 0-100 | 100-200 | 200-300 | >300 |  |  |  | Per 100 g/day |  |
| Male | 1.00 (reference) | 0.98 (0.88, 1.09) | 1.00 (0.89, 1.13) | 0.99 (0.84, 1.16) | 0.85 (0.67, 1.06) |  |  | 0.39 | 0.98 (0.94, 1.02) | 0.33 |
| Female | 1.00 (reference) | 0.96 (0.87, 1.06) | 0.87 (0.77, 0.97) | 0.90 (0.77, 1.06) | 0.91 (0.72, 1.15) |  |  | 0.03 | 0.96 (0.92, 1.00) |  |
| Cheese (g/day) | 0-20 | 20-40 | 40-60 | 60-80 | 80-100 | >100 |  |  | Per 10 g/day |  |
| Male | 1.00 (reference) | 1.03 (0.91, 1.16) | 1.12 (0.98, 1.28) | 1.11 (0.94, 1.30) | 1.04 (0.85, 1.27) | 1.49 (1.23, 1.81) |  | <0.01 | 1.02 (1.01, 1.04) | 0.01 |
| Female | 1.00 (reference) | 0.92 (0.82, 1.02) | 0.95 (0.84, 1.08) | 0.96 (0.82, 1.12) | 0.89 (0.73, 1.09) | 1.02 (0.84, 1.25) |  | 0.87 | 1.00 (0.99, 1.01) |  |
| Cream (g/day) | 0-10 | 10-20 | 20-30 | 30-40 | 40-50 | >50 |  |  | Per 10 g/day |  |
| Male | 1.00 (reference) | 1.01 (0.91, 1.12) | 0.94 (0.82, 1.08) | 0.81 (0.67, 0.98) | 0.89 (0.70, 1.13) | 0.76 (0.60, 0.96) |  | <0.01 | 0.96 (0.93, 0.98) | 0.46 |
| Female | 1.00 (reference) | 0.83 (0.76, 0.92) | 0.82 (0.72, 0.93) | 0.75 (0.62, 0.92) | 0.87 (0.68, 1.13) | 0.81 (0.62, 1.05) |  | <0.001 | 0.94 (0.91, 0.97) |  |
| Butter (g/day) | 0 | 0-10 | 10-20 | 20-30 | 30-40 | 40-50 | >50 |  | Per 10 g/day |  |
| Male | 1.00 (reference) | 0.91 (0.78, 1.05) | 0.93 (0.79, 1.11) | 0.88 (0.72, 1.06) | 0.84 (0.68, 1.04) | 0.88 (0.69, 1.13) | 0.91 (0.77, 1.07) | 0.05 | 0.98 (0.96, 1.00) | 0.06 |
| Female | 1.00 (reference) | 0.91 (0.81, 1.02) | 0.94 (0.81, 1.09) | 0.81 (0.68, 0.96) | 0.88 (0.72, 1.08) | 0.77 (0.57, 1.03) | 0.64 (0.49, 0.83) | <0.0001 | 0.95 (0.92, 0.97) |  |
| ^1^ Values are given as hazard ratios and 95% confidence intervals within parentheses, calculated using Cox models. | | | | | | | | | | |
| Adjusted for age, dietary assessment version (method), season, total energy intake, leisure-time physical activity, alcohol consumption, smoking status, educational level, family history of diabetes, lipid-lowering medication, hypertension at baseline, personal history of cardiovascular disease, personal history of cancer, fiber, vegetable and fruit, meat, soft drinks, and coffee. | | | | | | | | | | |
|  |  |  |  |  |  |  |  |  |  |  |

| **Supplementary Table 7.** Associations between dairy products and risk of type 2 diabetes, with additional adjustment for carbohydrates (n=26,461) ^1^ | | | | | | | | | |  |
| --- | --- | --- | --- | --- | --- | --- | --- | --- | --- | --- |
|  | Intake categories | | | | | | | *P* for trend | Continuous |  |
|  | 1 | 2 | 3 | 4 | 5 | 6 | 7 |  |  |  |
| Non-fermented milk (g/day) | 0-200 | 200-400 | 400-600 | 600-800 | 800-1000 | >1000 |  |  | Per 100 g/day |  |
| Number of participants | 11,789 | 8,072 | 4,204 | 1,499 | 501 | 396 |  |  |  |  |
| Number of cases | 1,883 | 1,382 | 780 | 304 | 112 | 91 |  |  |  |  |
| Person-years | 248,045 | 165,431 | 83,154 | 28,573 | 9,457 | 6,891 |  |  |  |  |
| Incidence per 1000 person-years | 7.59 | 8.35 | 9.38 | 10.64 | 11.84 | 13.21 |  |  |  |  |
| Model 1 | 1.00 (reference) | 1.09 (1.01, 1.17) | 1.18 (1.08, 1.29) | 1.27 (1.12, 1.44) | 1.47 (1.21, 1.79) | 1.42 (1.14, 1.77) |  | <0.0001 | 1.04 (1.03, 1.05) |  |
| Model 2 | 1.00 (reference) | 1.05 (0.98, 1.12) | 1.10 (1.01, 1.20) | 1.11 (0.98, 1.26) | 1.25 (1.02, 1.52) | 1.15 (0.93, 1.44) |  | <0.01 | 1.02 (1.01, 1.03) |  |
| Fermented milk (g/day) | 0 | 0-100 | 100-200 | 200-300 | >300 |  |  |  | Per 100 g/day |  |
| Number of participants | 9,239 | 7,982 | 5,766 | 2,405 | 1,069 |  |  |  |  |  |
| Number of cases | 1,760 | 1,340 | 914 | 377 | 161 |  |  |  |  |  |
| Person-years | 180,320 | 166,221 | 121,657 | 50,710 | 22,641 |  |  |  |  |  |
| Incidence per 1000 person-years | 9.76 | 8.06 | 7.51 | 7.43 | 7.11 |  |  |  |  |  |
| Model 1 | 1.00 (reference) | 0.97 (0.90, 1.04) | 0.93 (0.85, 1.01) | 0.94 (0.84, 1.06) | 0.88 (0.75, 1.04) |  |  | 0.04 | 0.97 (0.94, 1.00) |  |
| Model 2 | 1.00 (reference) | 0.97 (0.90, 1.04) | 0.93 (0.86, 1.01) | 0.98 (0.87, 1.09) | 0.90 (0.76, 1.06) |  |  | 0.12 | 0.98 (0.95, 1.01) |  |
| Cheese (g/day) | 0-20 | 20-40 | 40-60 | 60-80 | 80-100 | >100 |  |  | Per 10 g/day |  |
| Number of participants | 5,611 | 8,177 | 6,099 | 3,231 | 1,681 | 1,662 |  |  |  |  |
| Number of cases | 1,028 | 1,386 | 1,037 | 538 | 265 | 298 |  |  |  |  |
| Person-years | 107,237 | 166,817 | 126,915 | 68,773 | 36,374 | 35,434 |  |  |  |  |
| Incidence per 1000 person-years | 9.59 | 8.31 | 8.17 | 7.82 | 7.29 | 8.41 |  |  |  |  |
| Model 1 | 1.00 (reference) | 0.96 (0.89, 1.05) | 1.02 (0.93, 1.12) | 1.01 (0.91, 1.13) | 0.95 (0.82, 1.09) | 1.22 (1.05, 1.40) |  | 0.08 | 1.01 (1.00, 1.02) |  |
| Model 2 | 1.00 (reference) | 0.96 (0.89, 1.05) | 1.02 (0.93, 1.11) | 1.00 (0.89, 1.12) | 0.93 (0.80, 1.07) | 1.15 (1.00, 1.33) |  | 0.27 | 1.01 (0.99, 1.02) |  |
| Cream (g/day) | 0-10 | 10-20 | 20-30 | 30-40 | 40-50 | >50 |  |  | Per 10 g/day |  |
| Number of participants | 12,636 | 6,995 | 3,434 | 1,639 | 840 | 917 |  |  |  |  |
| Number of cases | 2,361 | 1,140 | 549 | 235 | 133 | 134 |  |  |  |  |
| Person-years | 254,793 | 144,986 | 71,496 | 34,395 | 17,007 | 18,874 |  |  |  |  |
| Incidence per 1000 person-years | 9.27 | 7.86 | 7.68 | 6.83 | 7.82 | 7.10 |  |  |  |  |
| Model 1 | 1.00 (reference) | 0.91 (0.84, 0.97) | 0.87 (0.79, 0.96) | 0.77 (0.67, 0.88) | 0.87 (0.73, 1.03) | 0.76 (0.64, 0.91) |  | <0.0001 | 0.95 (0.93, 0.97) |  |
| Model 2 | 1.00 (reference) | 0.94 (0.88, 1.01) | 0.92 (0.84, 1.01) | 0.83 (0.73, 0.96) | 0.93 (0.78, 1.11) | 0.84 (0.70, 1.00) |  | <0.01 | 0.97 (0.95, 0.99) |  |
| Butter (g/day) | 0 | 0-10 | 10-20 | 20-30 | 30-40 | 40-50 | >50 |  | Per 10 g/day |  |
| Number of participants | 15,049 | 3,687 | 2,192 | 1,787 | 1,351 | 772 | 1,623 |  |  |  |
| Number of cases | 2,794 | 572 | 353 | 260 | 204 | 118 | 251 |  |  |  |
| Person-years | 306,400 | 78,193 | 45,275 | 36,981 | 27,142 | 15,790 | 31,769 |  |  |  |
| Incidence per 1000 person-years | 9.12 | 7.32 | 7.80 | 7.03 | 7.52 | 7.47 | 7.90 |  |  |  |
| Model 1 | 1.00 (reference) | 0.90 (0.83, 0.99) | 0.94 (0.84, 1.05) | 0.83 (0.73, 0.95) | 0.85 (0.73, 0.98) | 0.82 (0.68, 0.98) | 0.79 (0.69, 0.91) | <0.0001 | 0.96 (0.95, 0.98) |  |
| Model 2 | 1.00 (reference) | 0.92 (0.84, 1.01) | 0.97 (0.87, 1.08) | 0.87 (0.77, 0.99) | 0.90 (0.78, 1.04) | 0.92 (0.76, 1.11) | 0.86 (0.75, 0.99) | <0.01 | 0.98 (0.96, 0.99) |  |
| ^1^ Values are given as hazard ratios and 95% confidence intervals within parentheses, calculated using Cox models. | | | | | | | | | |  |
| Model 1: adjusted for age, sex, dietary assessment version (method), season, total energy intake, leisure-time physical activity, alcohol consumption, smoking status, educational level, family history of diabetes, lipid-lowering medication, hypertension at baseline, personal history of cardiovascular disease, personal history of cancer, fiber, vegetable and fruit, meat, soft drinks, coffee, and percent total energy from carbohydrates. | | | | | | | | | |  |
|  |  |  |  |  |  |  |  |  |  |  |
|  |  |  |  |  |  |  |  |  |  |  |
| Model 2: adjusted for variables in model 1 plus body mass index. | | | | | | | | | |  |

| **Supplementary Table 8.** Association between dairy intake and risk of type 2 diabetes, excluding participants with prevalent cardiovascular disease or cancer at baseline (n=24,152) ^1^ | | | | | | | | | |
| --- | --- | --- | --- | --- | --- | --- | --- | --- | --- |
|  | Intake categories | | | | | | | *P* for trend | Continuous |
|  | 1 | 2 | 3 | 4 | 5 | 6 | 7 |  |  |
| Non-fermented milk (g/day) | 0-200 | 200-400 | 400-600 | 600-800 | 800-1000 | >1000 |  |  | Per 100 g/day |
| Number of participants | 10,770 | 7,382 | 3,816 | 1,375 | 451 | 358 |  |  |  |
| Number of cases | 1,716 | 1,255 | 713 | 277 | 101 | 84 |  |  |  |
| Person-years | 230,224 | 153,846 | 77,025 | 26,775 | 8,667 | 6,347 |  |  |  |
| Incidence per 1000 person-years | 7.45 | 8.16 | 9.26 | 10.35 | 11.65 | 13.23 |  |  |  |
| Model 1 | 1.00 (reference) | 1.12 (1.04, 1.21) | 1.28 (1.17, 1.40) | 1.42 (1.25, 1.62) | 1.61 (1.31, 1.97) | 1.77 (1.42, 2.22) |  | <0.0001 | 1.06 (1.05, 1.07) |
| Model 2 | 1.00 (reference) | 1.09 (1.01, 1.17) | 1.20 (1.10, 1.32) | 1.28 (1.13, 1.47) | 1.43 (1.17, 1.76) | 1.48 (1.18, 1.86) |  | <0.0001 | 1.04 (1.03, 1.06) |
| Model 3 | 1.00 (reference) | 1.07 (1.00, 1.16) | 1.18 (1.08, 1.30) | 1.25 (1.09, 1.42) | 1.44 (1.17, 1.77) | 1.45 (1.15, 1.82) |  | <0.0001 | 1.04 (1.03, 1.05) |
| Model 4 | 1.00 (reference) | 1.03 (0.96, 1.11) | 1.10 (1.01, 1.21) | 1.10 (0.97, 1.26) | 1.24 (1.01, 1.52) | 1.20 (0.96, 1.51) |  | <0.01 | 1.02 (1.01, 1.04) |
| Fermented milk (g/day) | 0 | 0-100 | 100-200 | 200-300 | >300 |  |  |  | Per 100 g/day |
| Number of participants | 8,391 | 7,335 | 5,276 | 2,179 | 971 |  |  |  |  |
| Number of cases | 1,589 | 1,237 | 832 | 348 | 140 |  |  |  |  |
| Person-years | 167,045 | 155,005 | 112,932 | 46,815 | 21,087 |  |  |  |  |
| Incidence per 1000 person-years | 9.51 | 7.98 | 7.37 | 7.43 | 6.64 |  |  |  |  |
| Model 1 | 1.00 (reference) | 0.92 (0.85, 0.99) | 0.84 (0.77, 0.91) | 0.83 (0.74, 0.93) | 0.73 (0.61, 0.87) |  |  | <0.0001 | 0.92 (0.90, 0.95) |
| Model 2 | 1.00 (reference) | 0.97 (0.90, 1.05) | 0.91 (0.84, 0.99) | 0.91 (0.81, 1.02) | 0.79 (0.67, 0.95) |  |  | <0.01 | 0.95 (0.92, 0.98) |
| Model 3 | 1.00 (reference) | 0.98 (0.91, 1.06) | 0.93 (0.86, 1.02) | 0.96 (0.86, 1.08) | 0.84 (0.70, 1.00) |  |  | 0.04 | 0.97 (0.94, 1.00) |
| Model 4 | 1.00 (reference) | 0.98 (0.90, 1.05) | 0.94 (0.86, 1.02) | 0.99 (0.88, 1.11) | 0.86 (0.72, 1.03) |  |  | 0.12 | 0.98 (0.95, 1.01) |
| Cheese (g/day) | 0-20 | 20-40 | 40-60 | 60-80 | 80-100 | >100 |  |  | Per 10 g/day |
| Number of participants | 4,974 | 7,424 | 5,636 | 2,990 | 1,583 | 1,545 |  |  |  |
| Number of cases | 917 | 1,254 | 953 | 498 | 250 | 274 |  |  |  |
| Person-years | 97,239 | 154,112 | 119,096 | 64,513 | 34,600 | 33,324 |  |  |  |
| Incidence per 1000 person-years | 9.43 | 8.14 | 8.00 | 7.72 | 7.23 | 8.22 |  |  |  |
| Model 1 | 1.00 (reference) | 0.88 (0.81, 0.96) | 0.89 (0.81, 0.97) | 0.86 (0.77, 0.96) | 0.81 (0.70, 0.93) | 0.95 (0.82, 1.10) |  | 0.06 | 0.99 (0.98, 1.00) |
| Model 2 | 1.00 (reference) | 0.94 (0.86, 1.02) | 0.96 (0.87, 1.06) | 0.95 (0.85, 1.07) | 0.90 (0.78, 1.04) | 1.06 (0.92, 1.23) |  | 0.96 | 1.00 (0.99, 1.01) |
| Model 3 | 1.00 (reference) | 0.95 (0.87, 1.04) | 1.01 (0.92, 1.11) | 1.01 (0.90, 1.13) | 0.94 (0.82, 1.09) | 1.20 (1.04, 1.39) |  | 0.08 | 1.01 (1.00, 1.02) |
| Model 4 | 1.00 (reference) | 0.95 (0.87, 1.03) | 1.00 (0.91, 1.10) | 0.98 (0.87, 1.10) | 0.91 (0.78, 1.05) | 1.10 (0.95, 1.28) |  | 0.57 | 1.00 (0.99, 1.01) |
| Cream (g/day) | 0-10 | 10-20 | 20-30 | 30-40 | 40-50 | >50 |  |  | Per 10 g/day |
| Number of participants | 11,531 | 6,394 | 3,117 | 1,502 | 766 | 842 |  |  |  |
| Number of cases | 2,153 | 1,039 | 494 | 217 | 124 | 119 |  |  |  |
| Person-years | 236,681 | 134,774 | 65,887 | 32,157 | 15,771 | 17,613 |  |  |  |
| Incidence per 1000 person-years | 9.10 | 7.71 | 7.50 | 6.75 | 7.86 | 6.76 |  |  |  |
| Model 1 | 1.00 (reference) | 0.86 (0.80, 0.92) | 0.83 (0.75, 0.92) | 0.73 (0.64, 0.84) | 0.84 (0.70, 1.01) | 0.71 (0.59, 0.86) |  | <0.0001 | 0.93 (0.91, 0.95) |
| Model 2 | 1.00 (reference) | 0.90 (0.83, 0.97) | 0.87 (0.79, 0.96) | 0.78 (0.67, 0.89) | 0.88 (0.73, 1.05) | 0.73 (0.60, 0.88) |  | <0.0001 | 0.94 (0.92, 0.97) |
| Model 3 | 1.00 (reference) | 0.90 (0.84, 0.98) | 0.87 (0.79, 0.96) | 0.78 (0.68, 0.90) | 0.88 (0.74, 1.06) | 0.74 (0.62, 0.90) |  | <0.0001 | 0.95 (0.93, 0.97) |
| Model 4 | 1.00 (reference) | 0.94 (0.87, 1.01) | 0.92 (0.83, 1.01) | 0.84 (0.73, 0.97) | 0.94 (0.78, 1.13) | 0.81 (0.67, 0.97) |  | <0.01 | 0.96 (0.94, 0.98) |
| Butter (g/day) | 0 | 0-10 | 10-20 | 20-30 | 30-40 | 40-50 | >50 |  | Per 10 g/day |
| Number of participants | 13,634 | 3,386 | 2,004 | 1,652 | 1,268 | 710 | 1,498 |  |  |
| Number of cases | 2,519 | 526 | 325 | 244 | 192 | 109 | 231 |  |  |
| Person-years | 282,340 | 73,188 | 42,104 | 34,767 | 25,825 | 14,730 | 29,930 |  |  |
| Incidence per 1000 person-years | 8.92 | 7.19 | 7.72 | 7.02 | 7.43 | 7.40 | 7.72 |  |  |
| Model 1 | 1.00 (reference) | 0.84 (0.77, 0.93) | 0.89 (0.79, 1.00) | 0.81 (0.71, 0.93) | 0.84 (0.73, 0.98) | 0.82 (0.67, 0.99) | 0.83 (0.72, 0.96) | <0.0001 | 0.97 (0.95, 0.99) |
| Model 2 | 1.00 (reference) | 0.90 (0.82, 0.99) | 0.92 (0.82, 1.04) | 0.82 (0.72, 0.94) | 0.83 (0.72, 0.97) | 0.79 (0.65, 0.96) | 0.78 (0.68, 0.90) | <0.0001 | 0.96 (0.95, 0.98) |
| Model 3 | 1.00 (reference) | 0.91 (0.83, 1.00) | 0.96 (0.85, 1.07) | 0.85 (0.75, 0.97) | 0.86 (0.75, 1.00) | 0.84 (0.69, 1.02) | 0.81 (0.70, 0.94) | <0.001 | 0.97 (0.95, 0.99) |
| Model 4 | 1.00 (reference) | 0.92 (0.83, 1.01) | 0.98 (0.87, 1.10) | 0.89 (0.78, 1.02) | 0.91 (0.78, 1.06) | 0.93 (0.76, 1.12) | 0.86 (0.74, 0.99) | 0.01 | 0.98 (0.96, 1.00) |
| ^1^ Values are given as hazard ratios and 95% confidence intervals within parentheses, calculated using Cox models. | | | | | | | | | |
| Model 1: adjusted for age, sex, dietary assessment version (method), season, and total energy intake. | | | | | | | | | |
| Model 2: adjusted for variables in model 1 plus leisure-time physical activity, alcohol consumption, smoking status, and educational level. | | | | | | | | | |
| Model 3: adjusted for variables in model 2 plus family history of diabetes, lipid-lowering medication, hypertension at baseline, fiber, vegetable and fruit, meat, soft drinks, and coffee. | | | | | | | | | |
| Model 4: adjusted for variables in model 3 plus body mass index. | | | | | | | | | |

| **Supplementary Table 9.** Association between dairy intake and risk of type 2 diabetes, excluding energy misreporters or those with substantial diet change (n=16,994) ^1^ | | | | | | | | | |
| --- | --- | --- | --- | --- | --- | --- | --- | --- | --- |
|  | Intake categories | | | | | | | *P* for trend | Continuous |
|  | 1 | 2 | 3 | 4 | 5 | 6 | 7 |  |  |
| Non-fermented milk (g/day) | 0-200 | 200-400 | 400-600 | 600-800 | 800-1000 | >1000 |  |  | Per 100 g/day |
| Number of participants | 7,312 | 5,351 | 2,803 | 965 | 320 | 243 |  |  |  |
| Number of cases | 1,034 | 843 | 484 | 177 | 69 | 56 |  |  |  |
| Person-years | 154,983 | 109,933 | 56,255 | 18,764 | 6,048 | 4,354 |  |  |  |
| Incidence per 1000 person-years | 6.67 | 7.67 | 8.60 | 9.43 | 11.41 | 12.86 |  |  |  |
| Model 1 | 1.00 (reference) | 1.14 (1.04, 1.25) | 1.26 (1.13, 1.41) | 1.32 (1.12, 1.55) | 1.58 (1.23, 2.02) | 1.67 (1.27, 2.20) |  | <0.0001 | 1.05 (1.04, 1.07) |
| Model 2 | 1.00 (reference) | 1.10 (1.01, 1.21) | 1.17 (1.04, 1.31) | 1.17 (0.99, 1.38) | 1.37 (1.07, 1.76) | 1.37 (1.04, 1.81) |  | <0.001 | 1.03 (1.02, 1.05) |
| Model 3 | 1.00 (reference) | 1.10 (1.00, 1.21) | 1.16 (1.04, 1.30) | 1.17 (0.99, 1.39) | 1.40 (1.09, 1.80) | 1.29 (0.97, 1.71) |  | <0.001 | 1.03 (1.01, 1.05) |
| Model 4 | 1.00 (reference) | 1.06 (0.97, 1.17) | 1.11 (0.99, 1.24) | 1.05 (0.89, 1.24) | 1.18 (0.92, 1.51) | 1.03 (0.78, 1.37) |  | 0.14 | 1.01 (1.00, 1.03) |
| Fermented milk (g/day) | 0 | 0-100 | 100-200 | 200-300 | >300 |  |  |  | Per 100 g/day |
| Number of participants | 5,902 | 5,111 | 3,788 | 1,555 | 638 |  |  |  |  |
| Number of cases | 1,032 | 762 | 562 | 222 | 85 |  |  |  |  |
| Person-years | 115,917 | 107,557 | 80,344 | 32,826 | 13,692 |  |  |  |  |
| Incidence per 1000 person-years | 8.90 | 7.08 | 6.99 | 6.76 | 6.21 |  |  |  |  |
| Model 1 | 1.00 (reference) | 0.87 (0.79, 0.96) | 0.85 (0.76, 0.94) | 0.79 (0.68, 0.91) | 0.70 (0.56, 0.87) |  |  | <0.0001 | 0.92 (0.88, 0.95) |
| Model 2 | 1.00 (reference) | 0.93 (0.85, 1.02) | 0.93 (0.84, 1.04) | 0.88 (0.76, 1.01) | 0.78 (0.62, 0.98) |  |  | <0.01 | 0.95 (0.91, 0.99) |
| Model 3 | 1.00 (reference) | 0.95 (0.86, 1.05) | 0.96 (0.87, 1.07) | 0.93 (0.80, 1.08) | 0.83 (0.66, 1.03) |  |  | 0.11 | 0.97 (0.93, 1.01) |
| Model 4 | 1.00 (reference) | 0.94 (0.86, 1.04) | 0.96 (0.86, 1.06) | 0.95 (0.82, 1.10) | 0.86 (0.69, 1.07) |  |  | 0.18 | 0.98 (0.94, 1.01) |
| Cheese (g/day) | 0-20 | 20-40 | 40-60 | 60-80 | 80-100 | >100 |  |  | Per 10 g/day |
| Number of participants | 3,156 | 5,359 | 4,088 | 2,213 | 1,133 | 1,045 |  |  |  |
| Number of cases | 483 | 819 | 648 | 354 | 176 | 183 |  |  |  |
| Person-years | 60,484 | 109,500 | 85,388 | 47,808 | 24,616 | 22,541 |  |  |  |
| Incidence per 1000 person-years | 7.99 | 7.48 | 7.59 | 7.40 | 7.15 | 8.12 |  |  |  |
| Model 1 | 1.00 (reference) | 0.94 (0.84, 1.06) | 0.97 (0.86, 1.09) | 0.94 (0.82, 1.08) | 0.88 (0.74, 1.06) | 1.02 (0.85, 1.22) |  | 0.70 | 1.00 (0.99, 1.01) |
| Model 2 | 1.00 (reference) | 1.01 (0.90, 1.13) | 1.06 (0.94, 1.20) | 1.06 (0.92, 1.22) | 1.00 (0.84, 1.20) | 1.16 (0.97, 1.40) |  | 0.17 | 1.01 (1.00, 1.02) |
| Model 3 | 1.00 (reference) | 1.05 (0.94, 1.18) | 1.14 (1.01, 1.28) | 1.13 (0.98, 1.31) | 1.09 (0.91, 1.30) | 1.33 (1.11, 1.60) |  | <0.01 | 1.02 (1.01, 1.03) |
| Model 4 | 1.00 (reference) | 1.04 (0.93, 1.17) | 1.12 (0.99, 1.27) | 1.10 (0.95, 1.26) | 1.02 (0.85, 1.22) | 1.24 (1.03, 1.49) |  | 0.06 | 1.01 (1.00, 1.02) |
| Cream (g/day) | 0-10 | 10-20 | 20-30 | 30-40 | 40-50 | >50 |  |  | Per 10 g/day |
| Number of participants | 7,423 | 4,699 | 2,458 | 1,177 | 591 | 646 |  |  |  |
| Number of cases | 1,215 | 720 | 370 | 170 | 94 | 94 |  |  |  |
| Person-years | 150,534 | 97,836 | 51,429 | 24,780 | 12,243 | 13,516 |  |  |  |
| Incidence per 1000 person-years | 8.07 | 7.36 | 7.19 | 6.86 | 7.68 | 6.95 |  |  |  |
| Model 1 | 1.00 (reference) | 0.92 (0.84, 1.01) | 0.88 (0.78, 0.99) | 0.81 (0.69, 0.95) | 0.88 (0.71, 1.08) | 0.77 (0.62, 0.95) |  | <0.001 | 0.95 (0.92, 0.97) |
| Model 2 | 1.00 (reference) | 0.97 (0.88, 1.06) | 0.92 (0.82, 1.03) | 0.86 (0.73, 1.01) | 0.92 (0.75, 1.14) | 0.78 (0.63, 0.97) |  | <0.01 | 0.96 (0.93, 0.98) |
| Model 3 | 1.00 (reference) | 0.97 (0.89, 1.07) | 0.92 (0.82, 1.03) | 0.85 (0.73, 1.00) | 0.95 (0.77, 1.18) | 0.80 (0.65, 0.99) |  | <0.01 | 0.96 (0.93, 0.98) |
| Model 4 | 1.00 (reference) | 1.02 (0.93, 1.12) | 0.96 (0.85, 1.08) | 0.92 (0.78, 1.09) | 1.02 (0.83, 1.26) | 0.88 (0.71, 1.09) |  | 0.22 | 0.98 (0.95, 1.00) |
| Butter (g/day) | 0 | 0-10 | 10-20 | 20-30 | 30-40 | 40-50 | >50 |  | Per 10 g/day |
| Number of participants | 9,043 | 2,371 | 1,505 | 1,293 | 995 | 590 | 1,197 |  |  |
| Number of cases | 1,508 | 334 | 220 | 184 | 145 | 91 | 181 |  |  |
| Person-years | 185,589 | 50,645 | 31,451 | 26,752 | 20,020 | 12,111 | 23,768 |  |  |
| Incidence per 1000 person-years | 8.13 | 6.59 | 6.99 | 6.88 | 7.24 | 7.51 | 7.62 |  |  |
| Model 1 | 1.00 (reference) | 0.86 (0.77, 0.97) | 0.90 (0.78, 1.03) | 0.88 (0.76, 1.03) | 0.88 (0.74, 1.05) | 0.86 (0.70, 1.06) | 0.80 (0.68, 0.94) | <0.001 | 0.97 (0.95, 0.99) |
| Model 2 | 1.00 (reference) | 0.92 (0.82, 1.04) | 0.93 (0.81, 1.07) | 0.89 (0.76, 1.04) | 0.87 (0.73, 1.03) | 0.83 (0.67, 1.03) | 0.74 (0.63, 0.87) | <0.0001 | 0.96 (0.94, 0.98) |
| Model 3 | 1.00 (reference) | 0.92 (0.82, 1.04) | 0.94 (0.82, 1.09) | 0.90 (0.77, 1.05) | 0.88 (0.74, 1.04) | 0.86 (0.69, 1.06) | 0.74 (0.63, 0.87) | <0.001 | 0.96 (0.94, 0.98) |
| Model 4 | 1.00 (reference) | 0.95 (0.84, 1.07) | 0.98 (0.85, 1.13) | 0.94 (0.81, 1.10) | 0.92 (0.77, 1.09) | 0.95 (0.77, 1.18) | 0.79 (0.67, 0.93) | 0.01 | 0.97 (0.96, 0.99) |
| ^1^ Values are given as hazard ratios and 95% confidence intervals within parentheses, calculated using Cox models. | | | | | | | | | |
| Model 1: adjusted for age, sex, dietary assessment version (method), season, and total energy intake. | | | | | | | | | |
| Model 2: adjusted for variables in model 1 plus leisure-time physical activity, alcohol consumption, smoking status, and educational level. | | | | | | | | | |
| Model 3: adjusted for variables in model 2 plus family history of diabetes, lipid-lowering medication, hypertension at baseline, personal history of cardiovascular disease, personal history of cancer, fiber, vegetable and fruit, meat, soft drinks, and coffee. | | | | | | | | | |
|  |  |  |  |  |  |  |  |  |  |
| Model 4: adjusted for variables in model 3 plus body mass index. | | | | | | | | | |

| **Supplementary Table 10.** Association between dairy intake and risk of type 2 diabetes, excluding type 2 diabetes cases that occurred within the first two years of follow-up (n=26,171) ^1^ | | | | | | | | | |
| --- | --- | --- | --- | --- | --- | --- | --- | --- | --- |
|  | Intake categories | | | | | | | *P* for trend | Continuous |
|  | 1 | 2 | 3 | 4 | 5 | 6 | 7 |  |  |
| Non-fermented milk (g/day) | 0-200 | 200-400 | 400-600 | 600-800 | 800-1000 | >1000 |  |  | Per 100 g/day |
| Number of participants | 11,687 | 7,979 | 4,151 | 1,480 | 489 | 385 |  |  |  |
| Number of cases | 1,781 | 1,289 | 727 | 285 | 100 | 80 |  |  |  |
| Person-years | 247,938 | 165,338 | 83,100 | 28,561 | 9,446 | 6,879 |  |  |  |
| Incidence per 1000 person-years | 7.18 | 7.80 | 8.75 | 9.98 | 10.59 | 11.63 |  |  |  |
| Model 1 | 1.00 (reference) | 1.11 (1.04, 1.20) | 1.26 (1.15, 1.38) | 1.43 (1.26, 1.63) | 1.53 (1.25, 1.88) | 1.63 (1.30, 2.06) |  | <0.0001 | 1.06 (1.04, 1.07) |
| Model 2 | 1.00 (reference) | 1.08 (1.01, 1.16) | 1.19 (1.08, 1.30) | 1.29 (1.13, 1.47) | 1.37 (1.11, 1.68) | 1.37 (1.09, 1.73) |  | <0.001 | 1.04 (1.03, 1.05) |
| Model 3 | 1.00 (reference) | 1.07 (1.00, 1.15) | 1.16 (1.06, 1.27) | 1.26 (1.11, 1.43) | 1.38 (1.12, 1.70) | 1.31 (1.04, 1.66) |  | <0.001 | 1.04 (1.02, 1.05) |
| Model 4 | 1.00 (reference) | 1.04 (0.97, 1.12) | 1.09 (1.00, 1.19) | 1.12 (0.98, 1.28) | 1.20 (0.98, 1.47) | 1.09 (0.87, 1.38) |  | 0.01 | 1.02 (1.01, 1.03) |
| Fermented milk (g/day) | 0 | 0-100 | 100-200 | 200-300 | >300 |  |  |  | Per 100 g/day |
| Number of participants | 9,132 | 7,887 | 5,712 | 2,379 | 1,061 |  |  |  |  |
| Number of cases | 1,653 | 1,245 | 860 | 351 | 153 |  |  |  |  |
| Person-years | 180,215 | 166,124 | 121,603 | 50,687 | 22,633 |  |  |  |  |
| Incidence per 1000 person-years | 9.17 | 7.49 | 7.07 | 6.92 | 6.76 |  |  |  |  |
| Model 1 | 1.00 (reference) | 0.89 (0.82, 0.95) | 0.83 (0.76, 0.90) | 0.80 (0.71, 0.89) | 0.77 (0.65, 0.90) |  |  | <0.0001 | 0.93 (0.90, 0.95) |
| Model 2 | 1.00 (reference) | 0.94 (0.87, 1.01) | 0.90 (0.83, 0.98) | 0.87 (0.78, 0.98) | 0.84 (0.71, 0.99) |  |  | <0.001 | 0.95 (0.93, 0.98) |
| Model 3 | 1.00 (reference) | 0.95 (0.88, 1.02) | 0.92 (0.84, 1.00) | 0.92 (0.82, 1.03) | 0.87 (0.74, 1.04) |  |  | 0.02 | 0.97 (0.94, 1.00) |
| Model 4 | 1.00 (reference) | 0.95 (0.88, 1.02) | 0.92 (0.85, 1.00) | 0.95 (0.85, 1.07) | 0.90 (0.76, 1.06) |  |  | 0.07 | 0.98 (0.95, 1.01) |
| Cheese (g/day) | 0-20 | 20-40 | 40-60 | 60-80 | 80-100 | >100 |  |  | Per 10 g/day |
| Number of participants | 5,526 | 8,090 | 6,032 | 3,207 | 1,670 | 1,646 |  |  |  |
| Number of cases | 943 | 1,299 | 970 | 514 | 254 | 282 |  |  |  |
| Person-years | 107,153 | 166,733 | 126,844 | 68,746 | 36,364 | 35,421 |  |  |  |
| Incidence per 1000 person-years | 8.80 | 7.79 | 7.65 | 7.48 | 6.98 | 7.96 |  |  |  |
| Model 1 | 1.00 (reference) | 0.90 (0.83, 0.98) | 0.90 (0.82, 0.99) | 0.89 (0.80, 0.99) | 0.83 (0.72, 0.96) | 0.98 (0.86, 1.13) |  | 0.17 | 0.99 (0.98, 1.00) |
| Model 2 | 1.00 (reference) | 0.96 (0.88, 1.04) | 0.98 (0.89, 1.07) | 0.98 (0.88, 1.10) | 0.92 (0.80, 1.07) | 1.10 (0.96, 1.27) |  | 0.55 | 1.00 (0.99, 1.01) |
| Model 3 | 1.00 (reference) | 0.98 (0.90, 1.07) | 1.04 (0.95, 1.14) | 1.05 (0.94, 1.17) | 0.98 (0.85, 1.14) | 1.25 (1.09, 1.45) |  | 0.02 | 1.01 (1.00, 1.02) |
| Model 4 | 1.00 (reference) | 0.98 (0.90, 1.06) | 1.02 (0.93, 1.12) | 1.02 (0.91, 1.14) | 0.95 (0.82, 1.10) | 1.17 (1.01, 1.35) |  | 0.16 | 1.01 (1.00, 1.02) |
| Cream (g/day) | 0-10 | 10-20 | 20-30 | 30-40 | 40-50 | >50 |  |  | Per 10 g/day |
| Number of participants | 12,468 | 6,928 | 3,408 | 1,626 | 832 | 909 |  |  |  |
| Number of cases | 2,193 | 1,073 | 523 | 222 | 125 | 126 |  |  |  |
| Person-years | 254,627 | 144,917 | 71,471 | 34,383 | 16,998 | 18,866 |  |  |  |
| Incidence per 1000 person-years | 8.61 | 7.40 | 7.32 | 6.46 | 7.35 | 6.68 |  |  |  |
| Model 1 | 1.00 (reference) | 0.87 (0.81, 0.94) | 0.86 (0.78, 0.94) | 0.74 (0.65, 0.85) | 0.84 (0.70, 1.00) | 0.75 (0.62, 0.90) |  | <0.0001 | 0.94 (0.92, 0.96) |
| Model 2 | 1.00 (reference) | 0.91 (0.85, 0.98) | 0.90 (0.81, 0.99) | 0.78 (0.68, 0.90) | 0.87 (0.72, 1.04) | 0.76 (0.64, 0.91) |  | <0.0001 | 0.95 (0.93, 0.97) |
| Model 3 | 1.00 (reference) | 0.92 (0.86, 0.99) | 0.90 (0.82, 0.99) | 0.79 (0.69, 0.91) | 0.89 (0.74, 1.07) | 0.79 (0.65, 0.94) |  | <0.0001 | 0.95 (0.93, 0.97) |
| Model 4 | 1.00 (reference) | 0.95 (0.88, 1.03) | 0.94 (0.86, 1.04) | 0.85 (0.74, 0.98) | 0.95 (0.79, 1.14) | 0.85 (0.71, 1.02) |  | 0.01 | 0.97 (0.95, 0.99) |
| Butter (g/day) | 0 | 0-10 | 10-20 | 20-30 | 30-40 | 40-50 | >50 |  | Per 10 g/day |
| Number of participants | 14,868 | 3,658 | 2,168 | 1,771 | 1,338 | 765 | 1,603 |  |  |
| Number of cases | 2,613 | 543 | 329 | 244 | 191 | 111 | 231 |  |  |
| Person-years | 306,216 | 78,160 | 45,254 | 36,967 | 27,128 | 15,783 | 31,754 |  |  |
| Incidence per 1000 person-years | 8.53 | 6.95 | 7.27 | 6.60 | 7.04 | 7.03 | 7.27 |  |  |
| Model 1 | 1.00 (reference) | 0.85 (0.78, 0.93) | 0.88 (0.78, 0.98) | 0.80 (0.70, 0.92) | 0.84 (0.72, 0.97) | 0.81 (0.67, 0.99) | 0.82 (0.71, 0.95) | <0.0001 | 0.97 (0.95, 0.98) |
| Model 2 | 1.00 (reference) | 0.91 (0.83, 0.99) | 0.91 (0.81, 1.02) | 0.81 (0.71, 0.92) | 0.83 (0.72, 0.96) | 0.79 (0.65, 0.95) | 0.77 (0.67, 0.89) | <0.0001 | 0.96 (0.94, 0.98) |
| Model 3 | 1.00 (reference) | 0.91 (0.83, 1.00) | 0.94 (0.83, 1.05) | 0.84 (0.74, 0.96) | 0.87 (0.75, 1.01) | 0.84 (0.69, 1.02) | 0.81 (0.70, 0.94) | <0.0001 | 0.97 (0.95, 0.99) |
| Model 4 | 1.00 (reference) | 0.93 (0.84, 1.02) | 0.96 (0.86, 1.08) | 0.88 (0.77, 1.00) | 0.91 (0.78, 1.06) | 0.93 (0.76, 1.12) | 0.86 (0.74, 0.99) | <0.01 | 0.98 (0.96, 0.99) |
| ^1^ Values are given as hazard ratios and 95% confidence intervals within parentheses, calculated using Cox models. | | | | | | | | | |
| Model 1: adjusted for age, sex, dietary assessment version (method), season, and total energy intake. | | | | | | | | | |
| Model 2: adjusted for variables in model 1 plus leisure-time physical activity, alcohol consumption, smoking status, and educational level. | | | | | | | | | |
| Model 3: adjusted for variables in model 2 plus family history of diabetes, lipid-lowering medication, hypertension at baseline, personal history of cardiovascular disease, personal history of cancer, fiber, vegetable and fruit, meat, soft drinks, and coffee. | | | | | | | | | |
|  |  |  |  |  |  |  |  |  |  |
| Model 4: adjusted for variables in model 3 plus body mass index. | | | | | | | | | |

| **Supplementary Table 11.** Associations between dairy products and risk of type 2 diabetes, with additional adjustment for socioeconomic classification index (n=26,461) ^1^ | | | | | | | | | |  |
| --- | --- | --- | --- | --- | --- | --- | --- | --- | --- | --- |
|  | Intake categories | | | | | | | *P* for trend | Continuous |  |
|  | 1 | 2 | 3 | 4 | 5 | 6 | 7 |  |  |  |
| Non-fermented milk (g/day) | 0-200 | 200-400 | 400-600 | 600-800 | 800-1000 | >1000 |  |  | Per 100 g/day |  |
| Number of participants | 11,789 | 8,072 | 4,204 | 1,499 | 501 | 396 |  |  |  |  |
| Number of cases | 1,883 | 1,382 | 780 | 304 | 112 | 91 |  |  |  |  |
| Person-years | 248,045 | 165,431 | 83,154 | 28,573 | 9,457 | 6,891 |  |  |  |  |
| Incidence per 1000 person-years | 7.59 | 8.35 | 9.38 | 10.64 | 11.84 | 13.21 |  |  |  |  |
| Model 1 | 1.00 (reference) | 1.08 (1.01, 1.16) | 1.17 (1.07, 1.27) | 1.25 (1.10, 1.42) | 1.45 (1.19, 1.76) | 1.38 (1.11, 1.72) |  | <0.0001 | 1.04 (1.03, 1.05) |  |
| Model 2 | 1.00 (reference) | 1.05 (0.97, 1.12) | 1.09 (1.00, 1.19) | 1.11 (0.98, 1.26) | 1.25 (1.02, 1.52) | 1.15 (0.92, 1.43) |  | <0.01 | 1.02 (1.01, 1.03) |  |
| Fermented milk (g/day) | 0 | 0-100 | 100-200 | 200-300 | >300 |  |  |  | Per 100 g/day |  |
| Number of participants | 9,239 | 7,982 | 5,766 | 2,405 | 1,069 |  |  |  |  |  |
| Number of cases | 1,760 | 1,340 | 914 | 377 | 161 |  |  |  |  |  |
| Person-years | 180,320 | 166,221 | 121,657 | 50,710 | 22,641 |  |  |  |  |  |
| Incidence per 1000 person-years | 9.76 | 8.06 | 7.51 | 7.43 | 7.11 |  |  |  |  |  |
| Model 1 | 1.00 (reference) | 0.97 (0.90, 1.05) | 0.93 (0.86, 1.01) | 0.94 (0.84, 1.06) | 0.88 (0.75, 1.04) |  |  | 0.04 | 0.97 (0.94, 1.00) |  |
| Model 2 | 1.00 (reference) | 0.97 (0.90, 1.04) | 0.94 (0.86, 1.02) | 0.98 (0.87, 1.09) | 0.90 (0.77, 1.07) |  |  | 0.13 | 0.98 (0.95, 1.01) |  |
| Cheese (g/day) | 0-20 | 20-40 | 40-60 | 60-80 | 80-100 | >100 |  |  | Per 10 g/day |  |
| Number of participants | 5,611 | 8,177 | 6,099 | 3,231 | 1,681 | 1,662 |  |  |  |  |
| Number of cases | 1,028 | 1,386 | 1,037 | 538 | 265 | 298 |  |  |  |  |
| Person-years | 107,237 | 166,817 | 126,915 | 68,773 | 36,374 | 35,434 |  |  |  |  |
| Incidence per 1000 person-years | 9.59 | 8.31 | 8.17 | 7.82 | 7.29 | 8.41 |  |  |  |  |
| Model 1 | 1.00 (reference) | 0.97 (0.90, 1.06) | 1.04 (0.95, 1.13) | 1.03 (0.92, 1.15) | 0.96 (0.84, 1.11) | 1.24 (1.08, 1.42) |  | 0.03 | 1.01 (1.00, 1.02) |  |
| Model 2 | 1.00 (reference) | 0.96 (0.89, 1.05) | 1.01 (0.93, 1.11) | 0.99 (0.89, 1.11) | 0.92 (0.80, 1.06) | 1.14 (0.99, 1.31) |  | 0.31 | 1.00 (0.99, 1.01) |  |
| Cream (g/day) | 0-10 | 10-20 | 20-30 | 30-40 | 40-50 | >50 |  |  | Per 10 g/day |  |
| Number of participants | 12,636 | 6,995 | 3,434 | 1,639 | 840 | 917 |  |  |  |  |
| Number of cases | 2,361 | 1,140 | 549 | 235 | 133 | 134 |  |  |  |  |
| Person-years | 254,793 | 144,986 | 71,496 | 34,395 | 17,007 | 18,874 |  |  |  |  |
| Incidence per 1000 person-years | 9.27 | 7.86 | 7.68 | 6.83 | 7.82 | 7.10 |  |  |  |  |
| Model 1 | 1.00 (reference) | 0.92 (0.85, 0.98) | 0.88 (0.80, 0.97) | 0.78 (0.68, 0.90) | 0.88 (0.74, 1.05) | 0.77 (0.65, 0.92) |  | <0.0001 | 0.95 (0.93, 0.97) |  |
| Model 2 | 1.00 (reference) | 0.94 (0.88, 1.01) | 0.92 (0.84, 1.01) | 0.84 (0.73, 0.96) | 0.93 (0.78, 1.11) | 0.84 (0.70, 1.00) |  | <0.01 | 0.97 (0.95, 0.99) |  |
| Butter (g/day) | 0 | 0-10 | 10-20 | 20-30 | 30-40 | 40-50 | >50 |  | Per 10 g/day |  |
| Number of participants | 15,049 | 3,687 | 2,192 | 1,787 | 1,351 | 772 | 1,623 |  |  |  |
| Number of cases | 2,794 | 572 | 353 | 260 | 204 | 118 | 251 |  |  |  |
| Person-years | 306,400 | 78,193 | 45,275 | 36,981 | 27,142 | 15,790 | 31,769 |  |  |  |
| Incidence per 1000 person-years | 9.12 | 7.32 | 7.80 | 7.03 | 7.52 | 7.47 | 7.90 |  |  |  |
| Model 1 | 1.00 (reference) | 0.91 (0.83, 1.00) | 0.94 (0.84, 1.05) | 0.84 (0.74, 0.96) | 0.86 (0.75, 1.00) | 0.84 (0.70, 1.01) | 0.82 (0.71, 0.94) | <0.0001 | 0.97 (0.95, 0.99) |  |
| Model 2 | 1.00 (reference) | 0.92 (0.84, 1.01) | 0.97 (0.87, 1.08) | 0.87 (0.77, 0.99) | 0.91 (0.78, 1.05) | 0.92 (0.77, 1.11) | 0.86 (0.75, 0.99) | <0.01 | 0.98 (0.96, 0.99) |  |
| ^1^ Values are given as hazard ratios and 95% confidence intervals within parentheses, calculated using Cox models. | | | | | | | | | |  |
| Model 1: adjusted for age, sex, dietary assessment version (method), season, total energy intake, leisure-time physical activity, alcohol consumption, smoking status, educational level, socioeconomic classification index, family history of diabetes, lipid-lowering medication, hypertension at baseline, personal history of cardiovascular disease, personal history of cancer, fiber, vegetable and fruit, meat, soft drinks, and coffee. | | | | | | | | | |  |
|  |  |  |  |  |  |  |  |  |  |  |
|  |  |  |  |  |  |  |  |  |  |  |
| Model 2: adjusted for variables in model 1 plus body mass index. | | | | | | | | | |  |

| **Supplementary Table 12.** E-values for associations between dairy products and risk of type 2 diabetes | | |
| --- | --- | --- |
| Dairy products | E-value for point estimate | E-value for 95% confidence interval closet to null |
| Non-fermented milk | 1.20 | 1.17 |
| Fermented milk | 1.17 | 1.00 |
| Cheese | 1.09 | 1.00 |
| Cream | 1.23 | 1.17 |
| Butter | 1.17 | 1.09 |
| ^1^ E-values were calculated using the publicly available online E-value calculator (https://www.evalue-calculator.com/evalue/). | | |

| **Supplementary Table 13.** Metabolite profiles for non-fermented milk identified from elastic net regression (45 metabolites) | | | | | | | | | | | | |
| --- | --- | --- | --- | --- | --- | --- | --- | --- | --- | --- | --- | --- |
| HMDB | CHEMICAL_NAME | SUPER_PATHWAY | SUB_PATHWAY | β^1^ | Unadjusted linear regression model^2^ | | | | Adjusted linear regression model^2^ | | | |
|  |  |  |  |  | *β* | 95% CI | | *P* | *β* | 95% CI | | *P* |
| HMDB0002064 | N-acetylputrescine | Amino Acid | Polyamine Metabolism | -0.0161 | -0.1905 | -0.3143 | -0.0668 | 0.0026 | -0.1673 | -0.2896 | -0.0450 | 0.0074 |
| HMDB0000123 | glycine | Amino Acid | Glycine, Serine and Threonine Metabolism | 0.0236 | 0.0121 | -0.0894 | 0.1136 | 0.8155 | 0.0054 | -0.1040 | 0.1148 | 0.9228 |
| HMDB0000167 | threonine | Amino Acid | Glycine, Serine and Threonine Metabolism | 0.0132 | 0.1235 | 0.0153 | 0.2318 | 0.0253 | 0.1270 | 0.0222 | 0.2317 | 0.0176 |
| HMDB0000407 | alpha-hydroxyisovalerate | Amino Acid | Leucine, Isoleucine and Valine Metabolism | -0.0193 | -0.1605 | -0.2655 | -0.0555 | 0.0028 | -0.1698 | -0.2826 | -0.0569 | 0.0032 |
| **HMDB0002366** | **tiglylcarnitine (C5:1-DC)** | **Amino Acid** | **Leucine, Isoleucine and Valine Metabolism** | **0.0836** | **0.2796** | **0.1688** | **0.3904** | **<0.0001** | **0.3057** | **0.1948** | **0.4165** | **<0.0001** |
| **HMDB0061115** | **tryptophan betaine** | **Amino Acid** | **Tryptophan Metabolism** | **-0.1142** | **-0.2548** | **-0.3564** | **-0.1531** | **<0.0001** | **-0.1739** | **-0.2761** | **-0.0716** | **0.0009** |
| **HMDB0001212** | **hydantoin-5-propionate** | **Amino Acid** | **Histidine Metabolism** | **0.0923** | **0.3362** | **0.2071** | **0.4653** | **<0.0001** | **0.3108** | **0.1764** | **0.4453** | **<0.0001** |
| HMDB0029432 | S-methylcysteine sulfoxide | Amino Acid | Methionine, Cysteine, SAM and Taurine Metabolism | -0.0383 | -0.2136 | -0.3136 | -0.1136 | <0.0001 | -0.1312 | -0.2311 | -0.0312 | 0.0102 |
| HMDB0000552 | 3-methylglutarylcarnitine (2) | Amino Acid | Leucine, Isoleucine and Valine Metabolism | 0.0235 | 0.1662 | 0.0507 | 0.2816 | 0.0048 | 0.1310 | 0.0117 | 0.2503 | 0.0315 |
|  | **N,N,N-trimethyl-5-aminovalerate** | **Amino Acid** | **Lysine Metabolism** | **0.1086** | **0.4371** | **0.3104** | **0.5639** | **<0.0001** | **0.3891** | **0.2629** | **0.5153** | **<0.0001** |
|  | N,N-dimethylalanine | Amino Acid | Alanine and Aspartate Metabolism | 0.0316 | 0.1104 | 0.0080 | 0.2129 | 0.0347 | 0.0857 | -0.0160 | 0.1874 | 0.0984 |
| **HMDB0000565** | **galactonate** | **Carbohydrate** | **Fructose, Mannose and Galactose Metabolism** | **0.1503** | **0.3051** | **0.1994** | **0.4107** | **<0.0001** | **0.2707** | **0.1680** | **0.3734** | **<0.0001** |
| HMDB0001851,HMDB0000568,HMDB0002917 | arabitol/xylitol | Carbohydrate | Pentose Metabolism | -0.0348 | -0.1169 | -0.2762 | 0.0425 | 0.1503 | -0.1201 | -0.2818 | 0.0415 | 0.1449 |
| HMDB0004193 | N1-Methyl-2-pyridone-5-carboxamide | Cofactors and Vitamins | Nicotinate and Nicotinamide Metabolism | 0.0018 | 0.2038 | 0.0931 | 0.3145 | 0.0003 | 0.1664 | 0.0547 | 0.2780 | 0.0036 |
| HMDB0240294 | 2-O-methylascorbic acid | Cofactors and Vitamins | Ascorbate and Aldarate Metabolism | -0.0174 | -0.1053 | -0.2322 | 0.0215 | 0.1035 | -0.0961 | -0.2303 | 0.0381 | 0.1602 |
|  | carotene diol (2) | Cofactors and Vitamins | Vitamin A Metabolism | -0.0997 | -0.2142 | -0.3160 | -0.1124 | <0.0001 | -0.1420 | -0.2521 | -0.0319 | 0.0115 |
| HMDB0002759 | androsterone sulfate | Lipid | Androgenic Steroids | 0.0312 | 0.1061 | -0.0097 | 0.2219 | 0.0724 | 0.0754 | -0.0446 | 0.1954 | 0.2176 |
|  | 1-linoleoyl-GPI (18:2)* | Lipid | Lysophospholipid | -0.0049 | -0.1424 | -0.2464 | -0.0384 | 0.0074 | -0.1145 | -0.2163 | -0.0127 | 0.0276 |
| HMDB0013078 | stearoyl ethanolamide | Lipid | Endocannabinoid | -0.0073 | -0.0877 | -0.2094 | 0.0340 | 0.1578 | -0.0738 | -0.1984 | 0.0509 | 0.2457 |
|  | eicosanedioate (C20-DC) | Lipid | Fatty Acid, Dicarboxylate | 0.0002 | -0.0106 | -0.1141 | 0.0929 | 0.8405 | -0.0325 | -0.1328 | 0.0678 | 0.5249 |
| HMDB0012097 | sphingomyelin (d18:1/14:0, d16:1/16:0)* | Lipid | Sphingomyelins | 0.0293 | 0.1720 | 0.0610 | 0.2830 | 0.0024 | 0.1327 | 0.0161 | 0.2492 | 0.0257 |
| HMDB0061666 | 2-hydroxyphytanate* | Lipid | Fatty Acid, Branched | -0.0297 | -0.1602 | -0.2650 | -0.0553 | 0.0028 | -0.1440 | -0.2477 | -0.0402 | 0.0066 |
|  | lignoceroyl sphingomyelin (d18:1/24:0) | Lipid | Sphingomyelins | -0.0061 | -0.1669 | -0.2817 | -0.0522 | 0.0044 | -0.1208 | -0.2345 | -0.0071 | 0.0374 |
|  | sphingomyelin (d17:1/16:0, d18:1/15:0, d16:1/17:0)* | Lipid | Sphingomyelins | 0.0279 | 0.1406 | 0.0355 | 0.2457 | 0.0088 | 0.0844 | -0.0256 | 0.1944 | 0.1324 |
|  | 1-palmitoleoylglycerol (16:1)* | Lipid | Monoacylglycerol | -0.0104 | -0.0702 | -0.1773 | 0.0369 | 0.1986 | -0.0790 | -0.1836 | 0.0256 | 0.1388 |
| HMDB0008123 | 1-oleoyl-2-docosahexaenoyl-GPC (18:1/22:6)* | Lipid | Phosphatidylcholine (PC) | 0.0008 | 0.0489 | -0.0664 | 0.1642 | 0.4050 | 0.0650 | -0.0573 | 0.1872 | 0.2974 |
| HMDB0002721 | N1-methylinosine | Nucleotide | Purine Metabolism, (Hypo)Xanthine/Inosine containing | 0.0897 | 0.2389 | 0.1095 | 0.3683 | 0.0003 | 0.2029 | 0.0667 | 0.3390 | 0.0035 |
| HMDB0000892 | pentose acid* | Partially Characterized Molecules | Partially Characterized Molecules | -0.0484 | -0.2253 | -0.3439 | -0.1068 | 0.0002 | -0.1569 | -0.2786 | -0.0352 | 0.0116 |
| HMDB0002994 | erythritol | Xenobiotics | Food Component/Plant | -0.0006 | -0.1154 | -0.2704 | 0.0396 | 0.1444 | -0.1321 | -0.2885 | 0.0243 | 0.0977 |
| HMDB0002123 | 1,3,7-trimethylurate | Xenobiotics | Xanthine Metabolism | -0.0007 | -0.1877 | -0.2942 | -0.0813 | 0.0006 | -0.1214 | -0.2291 | -0.0137 | 0.0273 |
| HMDB0011105 | 5-acetylamino-6-formylamino-3-methyluracil | Xenobiotics | Xanthine Metabolism | -0.0423 | -0.1820 | -0.2833 | -0.0808 | 0.0004 | -0.1504 | -0.2521 | -0.0488 | 0.0038 |
| HMDB0004400 | 5-acetylamino-6-amino-3-methyluracil | Xenobiotics | Xanthine Metabolism | -0.0103 | -0.1481 | -0.2523 | -0.0439 | 0.0054 | -0.1419 | -0.2519 | -0.0320 | 0.0115 |
| HMDB0013678 | 4-hydroxyhippurate | Xenobiotics | Benzoate Metabolism | -0.0611 | -0.1974 | -0.3241 | -0.0707 | 0.0023 | -0.1835 | -0.3068 | -0.0603 | 0.0036 |
| HMDB0012141 | 2,3-dihydroxyisovalerate | Xenobiotics | Food Component/Plant | -0.0178 | -0.1601 | -0.2758 | -0.0444 | 0.0067 | -0.0929 | -0.2113 | 0.0255 | 0.1238 |
|  | **3-bromo-5-chloro-2,6-dihydroxybenzoic acid*** | **Xenobiotics** | **Chemical** | **0.2460** | **0.4733** | **0.3525** | **0.5942** | **<0.0001** | **0.4519** | **0.3297** | **0.5742** | **<0.0001** |
|  | X - 12101 |  |  | -0.0062 | -0.1582 | -0.2829 | -0.0335 | 0.0130 | -0.1595 | -0.2836 | -0.0355 | 0.0118 |
|  | **X - 12798** |  |  | **0.0155** | **0.2693** | **0.1585** | **0.3801** | **<0.0001** | **0.2236** | **0.1159** | **0.3313** | **<0.0001** |
|  | X - 13507 |  |  | -0.0284 | -0.1229 | -0.2281 | -0.0177 | 0.0221 | -0.1178 | -0.2206 | -0.0151 | 0.0247 |
|  | X - 15728 |  |  | -0.0118 | -0.1441 | -0.2443 | -0.0440 | 0.0048 | -0.1548 | -0.2547 | -0.0548 | 0.0024 |
|  | X - 18901 |  |  | -0.0045 | -0.0732 | -0.1733 | 0.0269 | 0.1517 | -0.0751 | -0.1761 | 0.0259 | 0.1448 |
|  | **X - 18922** |  |  | **-0.0580** | **-0.1851** | **-0.2946** | **-0.0756** | **0.0009** | **-0.2411** | **-0.3505** | **-0.1317** | **<0.0001** |
|  | X - 21339 |  |  | -0.0376 | -0.1959 | -0.3019 | -0.0900 | 0.0003 | -0.1255 | -0.2313 | -0.0197 | 0.0202 |
|  | **X - 21736** |  |  | **-0.1273** | **-0.2042** | **-0.3166** | **-0.0918** | **0.0004** | **-0.2316** | **-0.3471** | **-0.1161** | **<0.0001** |
|  | X - 21834 |  |  | -0.0668 | -0.1370 | -0.2424 | -0.0316 | 0.0109 | -0.1327 | -0.2365 | -0.0289 | 0.0123 |
|  | **X - 23644** |  |  | **-0.0604** | **-0.2252** | **-0.3325** | **-0.1179** | **<0.0001** | **-0.1796** | **-0.2839** | **-0.0752** | **0.0008** |
| ^1^Beta coefficients were obtained from elastic net regression in the testing set. A total of 893 participants were randomized to either the training set (n=625) or the testing set (n=268) in a 7 to 3 fashion. | | | | | | | | | | | | |
| ^2^Linear regression was conducted in the overall sample (n=893). Multivariable linear regression models were adjusted for age, sex, season, total energy intake, leisure-time physical activity, alcohol consumption, smoking status, education, family history of diabetes, use of lipid-lowering drugs, hypertension at baseline, history of cardiovascular disease, history of cancer, fiber, vegetable and fruits, meat, soft drinks, coffee, and body mass index as well as baseline diabetes. The Bonferroni corrected *P* value was considered statistically significant. | | | | | | | | | | | | |
| The texts in bold indicate statistical significance in multivariable linear regression models after multiple corrections (*P*<0.05/45). | | | | | | | | | | | | |

| **Supplementary Table 14.** Metabolite profiles for fermented milk identified from elastic net regression (48 metabolites) | | | | | | | | | | | | |
| --- | --- | --- | --- | --- | --- | --- | --- | --- | --- | --- | --- | --- |
| HMDB | CHEMICAL_NAME | SUPER_PATHWAY | SUB_PATHWAY | β^1^ | Unadjusted linear regression model^2^ | | | | Adjusted linear regression model^2^ | | | |
|  |  |  |  |  | *β* | 95% CI | | *P* | *β* | 95% CI | | *P* |
| HMDB0000128 | guanidinoacetate | Amino Acid | Creatine Metabolism | -0.1200 | -0.6012 | -0.9341 | -0.2683 | 0.0004 | -0.3250 | -0.6756 | 0.0256 | 0.0692 |
| HMDB0000259 | serotonin | Amino Acid | Tryptophan Metabolism | 0.0429 | 0.5655 | 0.1402 | 0.9908 | 0.0092 | 0.6012 | 0.1759 | 1.0266 | 0.0056 |
| HMDB0000092 | dimethylglycine | Amino Acid | Glycine, Serine and Threonine Metabolism | -0.0446 | -0.4302 | -0.7807 | -0.0796 | 0.0162 | -0.1121 | -0.4672 | 0.2431 | 0.5359 |
|  | beta-hydroxyisovaleroylcarnitine | Amino Acid | Leucine, Isoleucine and Valine Metabolism | 0.1380 | 0.4909 | 0.1448 | 0.8369 | 0.0055 | 0.5628 | 0.2201 | 0.9055 | 0.0013 |
| HMDB0000996 | cysteine sulfinic acid | Amino Acid | Methionine, Cysteine, SAM and Taurine Metabolism | -0.0103 | -0.4511 | -0.7764 | -0.1258 | 0.0066 | -0.2941 | -0.6197 | 0.0316 | 0.0767 |
| HMDB0061384 | acisoga | Amino Acid | Polyamine Metabolism | -0.0536 | -0.4643 | -0.7986 | -0.1300 | 0.0065 | -0.3393 | -0.6707 | -0.0078 | 0.0449 |
| HMDB0062174 | methionine sulfone | Amino Acid | Methionine, Cysteine, SAM and Taurine Metabolism | 0.0490 | 0.7048 | 0.3355 | 1.0741 | 0.0002 | 0.4068 | 0.0177 | 0.7958 | 0.0405 |
| HMDB0000139,HMDB0006372 | glycerate | Carbohydrate | Glycolysis, Gluconeogenesis, and Pyruvate Metabolism | 0.0854 | 0.7718 | 0.4802 | 1.0634 | <0.0001 | 0.3115 | -0.0137 | 0.6367 | 0.0605 |
| **HMDB0000539** | **arabonate/xylonate** | **Carbohydrate** | **Pentose Metabolism** | **0.2420** | **0.8789** | **0.5101** | **1.2477** | **<0.0001** | **0.6829** | **0.2998** | **1.0661** | **0.0005** |
|  | lyxonate | Carbohydrate | Pentose Metabolism | 0.1116 | 0.8100 | 0.4662 | 1.1537 | <0.0001 | 0.4857 | 0.1331 | 0.8382 | 0.0070 |
| HMDB0002329 | oxalate (ethanedioate) | Cofactors and Vitamins | Ascorbate and Aldarate Metabolism | 0.2110 | 0.8729 | 0.5804 | 1.1653 | <0.0001 | 0.4231 | 0.0952 | 0.7511 | 0.0115 |
| HMDB0000855 | nicotinamide riboside | Cofactors and Vitamins | Nicotinate and Nicotinamide Metabolism | -0.1523 | -0.4348 | -0.7379 | -0.1317 | 0.0050 | -0.2211 | -0.5339 | 0.0918 | 0.1658 |
| HMDB0033844 | beta-cryptoxanthin | Cofactors and Vitamins | Vitamin A Metabolism | 0.0518 | 0.8330 | 0.5379 | 1.1282 | <0.0001 | 0.3358 | 0.0043 | 0.6674 | 0.0471 |
| HMDB0060649 | ascorbic acid 2-sulfate | Cofactors and Vitamins | Ascorbate and Aldarate Metabolism | 0.0344 | 0.3203 | 0.0273 | 0.6132 | 0.0322 | 0.1022 | -0.1889 | 0.3933 | 0.4909 |
| HMDB0001429 | phosphate | Energy | Oxidative Phosphorylation | 0.1256 | 0.5826 | 0.2761 | 0.8891 | 0.0002 | 0.3296 | 0.0096 | 0.6496 | 0.0435 |
| HMDB0000063 | cortisol | Lipid | Corticosteroids | 0.1504 | 0.3640 | -0.0283 | 0.7563 | 0.0689 | 0.3730 | -0.0170 | 0.7629 | 0.0608 |
| HMDB0002068 | erucate (22:1n9) | Lipid | Long Chain Monounsaturated Fatty Acid | -0.0036 | -0.2746 | -0.5813 | 0.0321 | 0.0793 | -0.1430 | -0.4544 | 0.1685 | 0.3678 |
| HMDB0000222 | palmitoylcarnitine (C16) | Lipid | Fatty Acid Metabolism (Acyl Carnitine, Long Chain Saturated) | -0.0249 | -0.4287 | -0.7456 | -0.1117 | 0.0081 | -0.0584 | -0.4049 | 0.2882 | 0.7411 |
| HMDB0000413 | 3-hydroxydodecanedioate* | Lipid | Fatty Acid, Dicarboxylate | -0.0053 | -0.4550 | -0.7563 | -0.1538 | 0.0031 | -0.2679 | -0.5764 | 0.0406 | 0.0886 |
| HMDB00653 | cholesterol sulfate | Lipid | Sterol | -0.0228 | -0.4335 | -0.7477 | -0.1193 | 0.0069 | -0.0954 | -0.4389 | 0.2481 | 0.5857 |
| HMDB0012383 | 1-stearoyl-2-arachidonoyl-GPS (18:0/20:4) | Lipid | Phosphatidylserine (PS) | 0.0166 | 0.2486 | -0.0447 | 0.5420 | 0.0966 | 0.3077 | 0.0183 | 0.5970 | 0.0372 |
|  | glycocholenate sulfate* | Lipid | Secondary Bile Acid Metabolism | -0.1439 | -0.6789 | -0.9884 | -0.3694 | <0.0001 | -0.3723 | -0.6935 | -0.0511 | 0.0231 |
| HMDB0240429,HMDB0186954 | androstenediol (3beta,17beta) monosulfate (2) | Lipid | Androgenic Steroids | 0.0156 | 0.2704 | -0.0600 | 0.6008 | 0.1086 | 0.0768 | -0.2602 | 0.4137 | 0.6550 |
|  | tridecenedioate (C13:1-DC)* | Lipid | Fatty Acid, Dicarboxylate | -0.0039 | -0.4189 | -0.7249 | -0.1129 | 0.0073 | -0.2843 | -0.5992 | 0.0306 | 0.0768 |
| HMDB0004980 | cis-4-decenoate (10:1n6)* | Lipid | Medium Chain Fatty Acid | -0.1016 | -0.2959 | -0.6182 | 0.0265 | 0.0720 | -0.3078 | -0.6313 | 0.0158 | 0.0623 |
|  | 17alpha-hydroxypregnanolone glucuronide | Lipid | Pregnenolone Steroids | -0.0386 | -0.5582 | -0.8608 | -0.2556 | 0.0003 | -0.1104 | -0.5018 | 0.2811 | 0.5802 |
|  | 3beta-hydroxy-5-cholestenoate | Lipid | Sterol | -0.0578 | -0.4593 | -0.7706 | -0.1480 | 0.0039 | -0.2496 | -0.5840 | 0.0848 | 0.1433 |
| HMDB0008946 | 1-palmitoyl-2-docosahexaenoyl-GPE (16:0/22:6)* | Lipid | Phosphatidylethanolamine (PE) | 0.0587 | 0.4481 | 0.1471 | 0.7491 | 0.0036 | 0.3447 | 0.0356 | 0.6539 | 0.0289 |
| HMDB0009078 | 1-oleoyl-2-docosahexaenoyl-GPE (18:1/22:6)* | Lipid | Phosphatidylethanolamine (PE) | 0.0751 | 0.3722 | 0.0762 | 0.6681 | 0.0138 | 0.2670 | -0.0290 | 0.5629 | 0.0770 |
|  | hexadecasphingosine (d16:1)* | Lipid | Sphingosines | 0.0330 | 0.3955 | 0.0934 | 0.6976 | 0.0104 | 0.3985 | 0.0959 | 0.7010 | 0.0099 |
|  | eicosenoylcarnitine (C20:1)* | Lipid | Fatty Acid Metabolism (Acyl Carnitine, Monounsaturated) | -0.0017 | -0.4481 | -0.7697 | -0.1265 | 0.0064 | -0.1958 | -0.5325 | 0.1409 | 0.2540 |
|  | sphingomyelin (d17:1/14:0, d16:1/15:0)* | Lipid | Sphingomyelins | 0.0324 | 0.7401 | 0.4223 | 1.0579 | <0.0001 | 0.3473 | -0.0239 | 0.7185 | 0.0666 |
|  | tetradecadienedioate (C14:2-DC)* | Lipid | Fatty Acid, Dicarboxylate | -0.0264 | -0.5759 | -0.8831 | -0.2686 | 0.0002 | -0.4829 | -0.7902 | -0.1756 | 0.0021 |
|  | decadienedioic acid (C10:2-DC)** | Lipid | Fatty Acid, Dicarboxylate | -0.0004 | -0.5461 | -0.8526 | -0.2396 | 0.0005 | -0.4792 | -0.7844 | -0.1741 | 0.0021 |
| HMDB0062775 | 4-vinylphenol sulfate | Xenobiotics | Benzoate Metabolism | -0.1023 | -0.6033 | -0.9006 | -0.3060 | <0.0001 | -0.2912 | -0.6389 | 0.0566 | 0.1006 |
|  | indolin-2-one | Xenobiotics | Food Component/Plant | 0.0122 | 0.3036 | -0.0178 | 0.6250 | 0.0640 | 0.2734 | -0.0442 | 0.5909 | 0.0914 |
|  | 4-hydroxychlorothalonil | Xenobiotics | Chemical | 0.2030 | 0.6310 | 0.2866 | 0.9753 | 0.0003 | 0.3205 | -0.0295 | 0.6704 | 0.0726 |
| HMDB0060018,HMDB0060016 | 1,2,3-benzenetriol sulfate (2) | Xenobiotics | Chemical | 0.0363 | 0.0587 | -0.2628 | 0.3801 | 0.7203 | 0.0898 | -0.2260 | 0.4056 | 0.5770 |
|  | (2,4 or 2,5)-dimethylphenol sulfate | Xenobiotics | Food Component/Plant | -0.1160 | -0.7355 | -1.0417 | -0.4293 | <0.0001 | -0.3681 | -0.7449 | 0.0087 | 0.0555 |
|  | 3,5-dichloro-2,6-dihydroxybenzoic acid | Xenobiotics | Chemical | 0.0429 | 0.5034 | 0.1976 | 0.8093 | 0.0013 | 0.3839 | 0.0611 | 0.7067 | 0.0198 |
|  | X - 11470 |  |  | -0.2959 | -0.6243 | -0.9834 | -0.2652 | 0.0007 | -0.4536 | -0.8084 | -0.0988 | 0.0123 |
|  | X - 12816 |  |  | -0.0093 | -0.5621 | -0.9036 | -0.2207 | 0.0013 | -0.6343 | -1.0333 | -0.2354 | 0.0019 |
|  | X - 13658 |  |  | -0.0015 | -0.4226 | -0.8164 | -0.0289 | 0.0354 | -0.2773 | -0.6718 | 0.1172 | 0.1681 |
|  | X - 21258 |  |  | 0.0273 | 0.3883 | 0.0820 | 0.6947 | 0.0130 | 0.2805 | -0.0245 | 0.5855 | 0.0715 |
|  | X - 21607 |  |  | -0.0123 | -0.3377 | -0.6463 | -0.0290 | 0.0321 | -0.2549 | -0.5628 | 0.0530 | 0.1046 |
|  | X - 21733 |  |  | 0.0835 | 0.6784 | 0.3768 | 0.9799 | <0.0001 | 0.3205 | -0.0070 | 0.6479 | 0.0551 |
|  | X - 23587 |  |  | -0.0421 | -0.3758 | -0.6849 | -0.0666 | 0.0173 | -0.3159 | -0.6233 | -0.0086 | 0.0440 |
|  | X - 25420 |  |  | -0.0136 | -0.5453 | -0.8579 | -0.2327 | 0.0006 | 0.0578 | -0.2990 | 0.4146 | 0.7506 |
| ^1^Beta coefficients were obtained from elastic net regression in the testing set. A total of 893 participants were randomized to either the training set (n=625) or the testing set (n=268) in a 7 to 3 fashion. | | | | | | | | | | | | |
| ^2^Linear regression was conducted in the overall sample (n=893). Multivariable linear regression models were adjusted for age, sex, season, total energy intake, leisure-time physical activity, alcohol consumption, smoking status, education, family history of diabetes, use of lipid-lowering drugs, hypertension at baseline, history of cardiovascular disease, history of cancer, fiber, vegetable and fruits, meat, soft drinks, coffee, and body mass index as well as baseline diabetes. The Bonferroni corrected *P* value was considered statistically significant. | | | | | | | | | | | | |
|  |  |  |  |  |  |  |  |  |  |  |  |  |
| The texts in bold indicate statistical significance in multivariable linear regression models after multiple corrections (*P*<0.05/48). | | | | | | | | | | | | |

| **Supplementary Table 15.** Metabolite profiles for cheese identified from elastic net regression (12 metabolites) | | | | | | | | | | | | |
| --- | --- | --- | --- | --- | --- | --- | --- | --- | --- | --- | --- | --- |
| HMDB | CHEMICAL_NAME | SUPER_PATHWAY | SUB_PATHWAY | β^1^ | Unadjusted linear regression model^2^ | | | | Adjusted linear regression model^2^ | | | |
|  |  |  |  |  | *β* | 95% CI | | *P* | *β* | 95% CI | | *P* |
| **HMDB0000092** | **dimethylglycine** | **Amino Acid** | **Glycine, Serine and Threonine Metabolism** | **-0.0671** | **-0.2299** | **-0.3466** | **-0.1131** | **0.0001** | **######** | **-0.3595** | **-0.1222** | **<0.0001** |
| HMDB0000479 | 3-methylhistidine | Amino Acid | Histidine Metabolism | 0.0229 | 0.1280 | 0.0209 | 0.2350 | 0.0192 | 0.1153 | 0.0088 | 0.2218 | 0.0339 |
|  | lyxonate | Carbohydrate | Pentose Metabolism | 0.0031 | 0.1733 | 0.0574 | 0.2891 | 0.0034 | 0.1218 | 0.0028 | 0.2408 | 0.0449 |
|  | **sphingomyelin (d17:1/16:0, d18:1/15:0, d16:1/17:0)*** | **Lipid** | **Sphingomyelins** | **0.0058** | **0.1570** | **0.0540** | **0.2600** | **0.0029** | **0.1625** | **0.0526** | **0.2725** | **0.0038** |
|  | **sphingomyelin (d18:1/21:0, d17:1/22:0, d16:1/23:0)*** | **Lipid** | **Sphingomyelins** | **0.0024** | **0.1743** | **0.0630** | **0.2856** | **0.0022** | **0.1896** | **0.0673** | **0.3119** | **0.0024** |
|  | **N-palmitoyl-heptadecasphingosine (d17:1/16:0)*** | **Lipid** | **Ceramides** | **0.0258** | **0.1673** | **0.0674** | **0.2673** | **0.0011** | **0.1525** | **0.0515** | **0.2535** | **0.0031** |
|  | sphingomyelin (d18:1/25:0, d19:0/24:1, d20:1/23:0, d19:1/24:0)* | Lipid | Sphingomyelins | 0.0056 | 0.1692 | 0.0635 | 0.2750 | 0.0017 | 0.1406 | 0.0325 | 0.2488 | 0.0109 |
| HMDB01448 | sulfate* | Xenobiotics | Chemical | 0.0044 | 0.1519 | 0.0206 | 0.2833 | 0.0235 | 0.1025 | -0.0325 | 0.2375 | 0.1364 |
|  | **N-methylpipecolate** | **Xenobiotics** | **Bacterial/Fungal** | **0.0492** | **0.1908** | **0.0883** | **0.2933** | **0.0003** | **0.1894** | **0.0877** | **0.2910** | **0.0003** |
|  | 3,5-dichloro-2,6-dihydroxybenzoic acid | Xenobiotics | Chemical | 0.0295 | 0.1537 | 0.0512 | 0.2562 | 0.0033 | 0.1087 | -0.0002 | 0.2176 | 0.0505 |
|  | X - 11381 |  |  | 0.0168 | 0.1596 | 0.0529 | 0.2663 | 0.0034 | 0.1354 | 0.0295 | 0.2412 | 0.0123 |
|  | X - 15461 |  |  | -0.0190 | -0.1572 | -0.2634 | -0.0511 | 0.0037 | -0.1115 | -0.2183 | -0.0047 | 0.0407 |
| ^1^Beta coefficients were obtained from elastic net regression in the testing set. A total of 893 participants were randomized to either the training set (n=625) or the testing set (n=268) in a 7 to 3 fashion. | | | | | | | | | | | | |
| ^2^Linear regression was conducted in the overall sample (n=893). Multivariable linear regression models were adjusted for age, sex, season, total energy intake, leisure-time physical activity, alcohol consumption, smoking status, education, family history of diabetes, use of lipid-lowering drugs, hypertension at baseline, history of cardiovascular disease, history of cancer, fiber, vegetable and fruits, meat, soft drinks, coffee, and body mass index as well as baseline diabetes. The Bonferroni corrected *P* value was considered statistically significant. | | | | | | | | | | | | |
| The texts in bold indicate statistical significance in multivariable linear regression models after multiple corrections (*P*<0.05/12). | | | | | | | | | | | | |

| **Supplementary Table 16.** Metabolite profiles for cream identified from elastic net regression (27 metabolites) | | | | | | | | | | | | |
| --- | --- | --- | --- | --- | --- | --- | --- | --- | --- | --- | --- | --- |
| HMDB | CHEMICAL_NAME | SUPER_PATHWAY | SUB_PATHWAY | β^1^ | Unadjusted linear regression model^2^ | | | | Adjusted linear regression model^2^ | | | |
|  |  |  |  |  | *β* | 95% CI | | *P* | *β* | 95% CI | | *P* |
| HMDB0000682 | 3-indoxyl sulfate | Amino Acid | Tryptophan Metabolism | 0.0152 | 0.1460 | -0.0182 | 0.3102 | 0.0813 | 0.1106 | -0.0539 | 0.2750 | 0.1872 |
| HMDB0061115 | tryptophan betaine | Amino Acid | Tryptophan Metabolism | 0.0174 | 0.1750 | 0.0169 | 0.3331 | 0.0301 | 0.1818 | 0.0208 | 0.3427 | 0.0269 |
| HMDB0240317 | N-formylphenylalanine | Amino Acid | Tyrosine Metabolism | -0.1522 | -0.2172 | -0.3883 | -0.0461 | 0.0129 | -0.1916 | -0.3617 | -0.0215 | 0.0273 |
| HMDB0006275 | dopamine 3-O-sulfate | Amino Acid | Tyrosine Metabolism | -0.0742 | -0.1954 | -0.3807 | -0.0100 | 0.0389 | -0.2386 | -0.4241 | -0.0530 | 0.0118 |
| HMDB0000699 | 1-methylnicotinamide | Cofactors and Vitamins | Nicotinate and Nicotinamide Metabolism | 0.0305 | 0.2831 | 0.1061 | 0.4600 | 0.0018 | 0.2195 | 0.0445 | 0.3945 | 0.0140 |
| HMDB0004194 | N1-Methyl-4-pyridone-3-carboxamide | Cofactors and Vitamins | Nicotinate and Nicotinamide Metabolism | 0.0625 | 0.1925 | 0.0303 | 0.3547 | 0.0201 | 0.2029 | 0.0428 | 0.3630 | 0.0131 |
| HMDB0000511 | caprate (10:0) | Lipid | Medium Chain Fatty Acid | 0.1345 | 0.3602 | 0.1766 | 0.5437 | 0.0001 | 0.2262 | 0.0379 | 0.4145 | 0.0186 |
| HMDB0061112 | 3-carboxy-4-methyl-5-propyl-2-furanpropanoate (CMPF) | Lipid | Fatty Acid, Dicarboxylate | 0.0071 | 0.2852 | 0.0946 | 0.4757 | 0.0034 | 0.2342 | 0.0338 | 0.4346 | 0.0220 |
| **HMDB0002829** | **androsterone glucuronide** | **Lipid** | **Androgenic Steroids** | **0.1651** | **0.2071** | **0.0432** | **0.3709** | **0.0133** | **0.2972** | **0.1137** | **0.4808** | **0.0015** |
| **HMDB0006210** | **margaroylcarnitine (C17)*** | **Lipid** | **Fatty Acid Metabolism (Acyl Carnitine, Long Chain Saturated)** | **0.0133** | **0.3844** | **0.2185** | **0.5503** | **<0.0001** | **0.2874** | **0.1161** | **0.4586** | **0.0010** |
|  | 2-hydroxylaurate | Lipid | Fatty Acid, Monohydroxy | 0.0042 | 0.1599 | -0.0021 | 0.3220 | 0.053 | 0.1577 | -0.0013 | 0.3167 | 0.0519 |
| HMDB0007883 | 1-myristoyl-2-arachidonoyl-GPC (14:0/20:4)* | Lipid | Phosphatidylcholine (PC) | 0.0310 | 0.1802 | 0.0090 | 0.3515 | 0.0392 | 0.1681 | -0.0102 | 0.3464 | 0.0645 |
|  | **sphingomyelin (d17:2/16:0, d18:2/15:0)*** | **Lipid** | **Sphingomyelins** | **0.1163** | **0.4042** | **0.2421** | **0.5663** | **<0.0001** | **0.4657** | **0.2731** | **0.6582** | **<0.0001** |
| HMDB0006455 | arachidonoylcarnitine (C20:4) | Lipid | Fatty Acid Metabolism (Acyl Carnitine, Polyunsaturated) | -0.0371 | -0.1928 | -0.3622 | -0.0234 | 0.0257 | -0.1548 | -0.3348 | 0.0252 | 0.0918 |
|  | dihomo-linolenoylcarnitine (C20:3n3 or 6)* | Lipid | Fatty Acid Metabolism (Acyl Carnitine, Polyunsaturated) | -0.0996 | -0.2109 | -0.3798 | -0.0421 | 0.0144 | -0.1642 | -0.3388 | 0.0104 | 0.0652 |
| HMDB0059745 | N-acetyl-2-aminooctanoate* | Lipid | Fatty Acid, Amino | 0.0134 | 0.2408 | 0.0679 | 0.4136 | 0.0064 | 0.2121 | 0.0338 | 0.3904 | 0.0198 |
|  | **sphingomyelin (d17:1/14:0, d16:1/15:0)*** | **Lipid** | **Sphingomyelins** | **0.2120** | **0.4644** | **0.2984** | **0.6303** | **<0.0001** | **0.4209** | **0.2269** | **0.6150** | **<0.0001** |
|  | branched chain 14:0 dicarboxylic acid** | Lipid | Fatty Acid, Dicarboxylate | -0.0282 | -0.1305 | -0.3002 | 0.0392 | 0.1317 | -0.2567 | -0.4336 | -0.0797 | 0.0045 |
|  | glutamine conjugate of C7H12O2* | Partially Characterized Molecules | Partially Characterized Molecules | 0.0147 | 0.2300 | 0.0619 | 0.3981 | 0.0074 | 0.1861 | 0.0122 | 0.3601 | 0.0360 |
| HMDB0001895 | salicylate | Xenobiotics | Drug - Topical Agents | -0.1063 | -0.2154 | -0.3980 | -0.0328 | 0.0208 | -0.1948 | -0.3767 | -0.0130 | 0.0357 |
| HMDB0001847 | caffeine | Xenobiotics | Xanthine Metabolism | 0.0388 | 0.0889 | -0.0836 | 0.2615 | 0.3121 | 0.0646 | -0.1106 | 0.2399 | 0.4694 |
|  | 4-hydroxycoumarin | Xenobiotics | Drug - Cardiovascular | -0.0158 | -0.0475 | -0.2110 | 0.1161 | 0.5692 | -0.0977 | -0.2624 | 0.0669 | 0.2444 |
|  | indolin-2-one | Xenobiotics | Food Component/Plant | 0.0174 | 0.1874 | 0.0188 | 0.3559 | 0.0294 | 0.1456 | -0.0218 | 0.3129 | 0.0882 |
| HMDB0029965 | methyl glucopyranoside (alpha + beta) | Xenobiotics | Food Component/Plant | -0.0457 | -0.1312 | -0.3138 | 0.0513 | 0.1586 | -0.2483 | -0.4390 | -0.0577 | 0.0107 |
| **HMDB0059586** | **perfluorooctanesulfonate (PFOS)** | **Xenobiotics** | **Chemical** | **0.1674** | **0.3984** | **0.2116** | **0.5852** | **<0.0001** | **0.3274** | **0.1286** | **0.5261** | **0.0013** |
| HMDB0059587 | perfluorooctanoate (PFOA) | Xenobiotics | Chemical | 0.0438 | 0.3574 | 0.1864 | 0.5284 | <0.0001 | 0.2654 | 0.0897 | 0.4411 | 0.0031 |
|  | X - 21285 |  |  | 0.0069 | 0.1058 | -0.0547 | 0.2663 | 0.1962 | 0.0565 | -0.1080 | 0.2209 | 0.5007 |
| ^1^Beta coefficients were obtained from elastic net regression in the testing set. A total of 893 participants were randomized to either the training set (n=625) or the testing set (n=268) in a 7 to 3 fashion. | | | | | | | | | | | | |
| ^2^Linear regression was conducted in the overall sample (n=893). Multivariable linear regression models were adjusted for age, sex, season, total energy intake, leisure-time physical activity, alcohol consumption, smoking status, education, family history of diabetes, use of lipid-lowering drugs, hypertension at baseline, history of cardiovascular disease, history of cancer, fiber, vegetable and fruits, meat, soft drinks, coffee, and body mass index as well as baseline diabetes. The Bonferroni corrected *P* value was considered statistically significant. | | | | | | | | | | | | |
|  |  |  |  |  |  |  |  |  |  |  |  |  |
| The texts in bold indicate statistical significance in multivariable linear regression models after multiple corrections (*P*<0.05/27). | | | | | | | | | | | | |

| **Supplementary Table 17.** Metabolite profiles for cream identified from elastic net regression (46 metabolites) | | | | | | | | | | | | |
| --- | --- | --- | --- | --- | --- | --- | --- | --- | --- | --- | --- | --- |
| HMDB | CHEMICAL_NAME | SUPER_PATHWAY | SUB_PATHWAY | β^1^ | Unadjusted linear regression model^2^ | | | | Adjusted linear regression model^2^ | | | |
|  |  |  |  |  | *β* | 95% CI | | *P* | *β* | 95% CI | | *P* |
| HMDB0000205 | phenylpyruvate | Amino Acid | Phenylalanine Metabolism | 0.0001 | 0.3204 | 0.0532 | 0.5875 | 0.0188 | 0.32265 | 0.06425 | 0.58105 | 0.0145 |
| HMDB0000670 | homoarginine | Amino Acid | Urea cycle; Arginine and Proline Metabolism | -0.0622 | -0.2197 | -0.4815 | 0.0421 | 0.1000 | -0.04131 | -0.30715 | 0.22453 | 0.7605 |
| HMDB0002302 | indolepropionate | Amino Acid | Tryptophan Metabolism | -0.0701 | -0.3840 | -0.6423 | -0.1258 | 0.0036 | -0.15375 | -0.42013 | 0.11263 | 0.2576 |
| HMDB0000866 | N-acetyltyrosine | Amino Acid | Tyrosine Metabolism | 0.0275 | 0.3071 | 0.0431 | 0.5712 | 0.0227 | 0.34021 | 0.0741 | 0.60633 | 0.0123 |
| HMDB0000459 | 3-methylcrotonylglycine | Amino Acid | Leucine, Isoleucine and Valine Metabolism | 0.0632 | 0.2200 | -0.0304 | 0.4704 | 0.0849 | 0.22513 | -0.01597 | 0.46624 | 0.0672 |
| **HMDB0000396** | **3-hydroxy-2-ethylpropionate** | **Amino Acid** | **Leucine, Isoleucine and Valine Metabolism** | **0.0793** | **0.7271** | **0.4692** | **0.9849** | **<0.0001** | **0.68964** | **0.42838** | **0.95089** | **<0.0001** |
|  | lanthionine | Amino Acid | Methionine, Cysteine, SAM and Taurine Metabolism | -0.2199 | -0.4292 | -0.7029 | -0.1555 | 0.0022 | -0.35101 | -0.61715 | -0.08488 | 0.0098 |
|  | N-acetyl-1-methylhistidine* | Amino Acid | Histidine Metabolism | 0.0155 | 0.2714 | -0.0315 | 0.5743 | 0.0790 | 0.2953 | -0.00473 | 0.59532 | 0.0537 |
| HMDB0002329 | oxalate (ethanedioate) | Cofactors and Vitamins | Ascorbate and Aldarate Metabolism | -0.0444 | -0.3729 | -0.6194 | -0.1264 | 0.0031 | -0.13283 | -0.39995 | 0.13429 | 0.3293 |
| HMDB0061717 | succinylcarnitine (C4-DC) | Energy | TCA Cycle | 0.0004 | 0.1201 | -0.1662 | 0.4063 | 0.4105 | 0.18222 | -0.10878 | 0.47322 | 0.2194 |
| HMDB0006270,HMDB0000673 | linoleate (18:2n6) | Lipid | Long Chain Polyunsaturated Fatty Acid (n3 and n6) | -0.1364 | -0.6631 | -0.9458 | -0.3803 | <0.0001 | -0.38281 | -0.66918 | -0.09643 | 0.0089 |
| HMDB0000626 | deoxycholate | Lipid | Secondary Bile Acid Metabolism | 0.0295 | 0.2463 | -0.0116 | 0.5042 | 0.0612 | 0.17701 | -0.07142 | 0.42545 | 0.1623 |
| HMDB0000039 | butyrate/isobutyrate (4:0) | Lipid | Short Chain Fatty Acid | 0.0126 | 0.3180 | 0.0603 | 0.5757 | 0.0156 | 0.3437 | 0.09731 | 0.5901 | 0.0063 |
| HMDB0000951 | taurochenodeoxycholate | Lipid | Primary Bile Acid Metabolism | 0.0489 | 0.4806 | 0.2097 | 0.7516 | 0.0005 | 0.34964 | 0.08737 | 0.61191 | 0.009 |
| HMDB0061714 | docosadienoate (22:2n6) | Lipid | Long Chain Polyunsaturated Fatty Acid (n3 and n6) | -0.1084 | -0.2496 | -0.5033 | 0.0042 | 0.0539 | -0.15594 | -0.40035 | 0.08847 | 0.2108 |
| HMDB0010383 | 1-palmitoleoyl-GPC (16:1)* | Lipid | Lysophospholipid | -0.0173 | -0.2255 | -0.5273 | 0.0764 | 0.1430 | -0.10229 | -0.39554 | 0.19096 | 0.4938 |
| HMDB0010395 | 1-arachidonoyl-GPC (20:4n6)* | Lipid | Lysophospholipid | -0.0130 | -0.0860 | -0.3533 | 0.1812 | 0.5277 | -0.04394 | -0.30624 | 0.21836 | 0.7424 |
| **HMDB0013205** | **cis-4-decenoylcarnitine (C10:1)** | **Lipid** | **Fatty Acid Metabolism (Acyl Carnitine, Monounsaturated)** | **-0.0024** | **-0.6532** | **-0.9310** | **-0.3753** | **<0.0001** | **-0.5686** | **-0.84433** | **-0.29287** | **<0.0001** |
| HMDB0000708 | glycoursodeoxycholate | Lipid | Secondary Bile Acid Metabolism | 0.0756 | 0.4153 | 0.1588 | 0.6719 | 0.0015 | 0.31647 | 0.0678 | 0.56514 | 0.0127 |
| **HMDB0006469** | **linoleoylcarnitine (C18:2)*** | **Lipid** | **Fatty Acid Metabolism (Acyl Carnitine, Polyunsaturated)** | **-0.1068** | **-0.6753** | **-0.9356** | **-0.4151** | **<0.0001** | **-0.54821** | **-0.81498** | **-0.28143** | **<0.0001** |
| **HMDB0006210** | **margaroylcarnitine (C17)*** | **Lipid** | **Fatty Acid Metabolism (Acyl Carnitine, Long Chain Saturated)** | **0.4904** | **0.8995** | **0.6404** | **1.1585** | **<0.0001** | **0.83745** | **0.57801** | **1.09689** | **<0.0001** |
| **HMDB0061666** | **2-hydroxyphytanate*** | **Lipid** | **Fatty Acid, Branched** | **0.1202** | **0.8721** | **0.6219** | **1.1222** | **<0.0001** | **0.72191** | **0.47482** | **0.96899** | **<0.0001** |
| HMDB0013288 | nonanoylcarnitine (C9) | Lipid | Fatty Acid Metabolism (Acyl Carnitine, Medium Chain) | 0.0965 | 0.4634 | 0.2105 | 0.7163 | 0.0003 | 0.34209 | 0.09402 | 0.59016 | 0.0069 |
| **HMDB0011342** | **1-(1-enyl-palmitoyl)-2-oleoyl-GPE (P-16:0/18:1)*** | **Lipid** | **Plasmalogen** | **0.1485** | **0.7221** | **0.4617** | **0.9825** | **<0.0001** | **0.66891** | **0.4159** | **0.92192** | **<0.0001** |
| **HMDB0008123** | **1-oleoyl-2-docosahexaenoyl-GPC (18:1/22:6)*** | **Lipid** | **Phosphatidylcholine (PC)** | **-0.4739** | **-0.9493** | **-1.2232** | **-0.6754** | **<0.0001** | **-0.80247** | **-1.09319** | **-0.51175** | **<0.0001** |
| **HMDB0007257** | **linoleoyl-arachidonoyl-glycerol (18:2/20:4) [1]*** | **Lipid** | **Diacylglycerol** | **-0.1064** | **-0.6690** | **-0.9203** | **-0.4177** | **<0.0001** | **-0.45056** | **-0.70407** | **-0.19705** | **0.0005** |
|  | **N-palmitoyl-heptadecasphingosine (d17:1/16:0)*** | **Lipid** | **Ceramides** | **0.1698** | **0.5304** | **0.2832** | **0.7775** | **<0.0001** | **0.46181** | **0.21914** | **0.70449** | **0.0002** |
| **HMDB0001348** | **sphingomyelin (d18:2/18:1)*** | **Lipid** | **Sphingomyelins** | **-0.2041** | **-1.0110** | **-1.3157** | **-0.7062** | **<0.0001** | **-0.95147** | **-1.2976** | **-0.60534** | **<0.0001** |
|  | dihomo-linolenoylcarnitine (C20:3n3 or 6)* | Lipid | Fatty Acid Metabolism (Acyl Carnitine, Polyunsaturated) | -0.0759 | -0.2702 | -0.5378 | -0.0026 | 0.0479 | -0.2719 | -0.54073 | -0.00306 | 0.0475 |
|  | **sphingomyelin (d17:1/14:0, d16:1/15:0)*** | **Lipid** | **Sphingomyelins** | **0.3353** | **0.5708** | **0.3063** | **0.8353** | **<0.0001** | **0.80867** | **0.51156** | **1.10578** | **<0.0001** |
|  | cis-3,4-methyleneheptanoylcarnitine | Lipid | Fatty Acid Metabolism (Acyl Carnitine, Medium Chain) | 0.0406 | -0.0858 | -0.3546 | 0.1830 | 0.5312 | -0.01571 | -0.2815 | 0.25008 | 0.9077 |
| HMDB0000462 | allantoin | Nucleotide | Purine Metabolism, (Hypo)Xanthine/Inosine containing | 0.0828 | 0.2258 | -0.0923 | 0.5439 | 0.164 | 0.19365 | -0.13237 | 0.51968 | 0.244 |
| HMDB0005923 | N4-acetylcytidine | Nucleotide | Pyrimidine Metabolism, Cytidine containing | 0.0333 | 0.2775 | 0.0060 | 0.5489 | 0.0451 | 0.26522 | -0.00020584 | 0.53065 | 0.0502 |
|  | 3-(3-amino-3-carboxypropyl)uridine* | Nucleotide | Pyrimidine Metabolism, Uracil containing | 0.0349 | 0.3148 | 0.0101 | 0.6196 | 0.0429 | 0.35236 | 0.05536 | 0.64937 | 0.0201 |
| **HMDB0000801** | **phytanate** | **Xenobiotics** | **Food Component/Plant** | **0.1801** | **0.9327** | **0.6768** | **1.1885** | **<0.0001** | **0.81126** | **0.55599** | **1.06654** | **<0.0001** |
| **HMDB0012141** | **2,3-dihydroxyisovalerate** | **Xenobiotics** | **Food Component/Plant** | **0.1087** | **0.6726** | **0.3932** | **0.9520** | **<0.0001** | **0.55639** | **0.27235** | **0.84042** | **0.0001** |
| HMDB0135245 | eugenol sulfate | Xenobiotics | Food Component/Plant | 0.0032 | 0.2172 | -0.0564 | 0.4909 | 0.1196 | 0.22923 | -0.03636 | 0.49481 | 0.0906 |
| **HMDB0240565** | **umbelliferone sulfate** | **Xenobiotics** | **Food Component/Plant** | **0.0175** | **0.4419** | **0.1896** | **0.6943** | **0.0006** | **0.43299** | **0.1851** | **0.68087** | **0.0006** |
|  | **X - 07765** |  |  | **-0.0450** | **-0.4726** | **-0.7359** | **-0.2093** | **0.0004** | **-0.43158** | **-0.68367** | **-0.17949** | **0.0008** |
|  | X - 11444 |  |  | 0.0470 | 0.3211 | -0.0322 | 0.6744 | 0.0748 | 0.44552 | 0.08639 | 0.80465 | 0.0151 |
|  | X - 12462 |  |  | -0.1168 | -0.3591 | -0.6616 | -0.0566 | 0.0200 | -0.22224 | -0.52406 | 0.07959 | 0.1488 |
|  | X - 13553 |  |  | 0.0433 | 0.2892 | -0.0075 | 0.5859 | 0.0560 | 0.35425 | 0.05463 | 0.65387 | 0.0205 |
|  | X - 21353 |  |  | -0.0033 | -0.4817 | -0.7413 | -0.2222 | 0.0003 | -0.35304 | -0.60955 | -0.09653 | 0.0070 |
|  | **X - 21383** |  |  | **-0.6150** | **-0.8873** | **-1.1534** | **-0.6212** | **<0.0001** | **-0.77215** | **-1.03488** | **-0.50942** | **<0.0001** |
|  | X - 24748 |  |  | 0.0498 | 0.3174 | 0.0568 | 0.5781 | 0.0171 | 0.34378 | 0.09563 | 0.59193 | 0.0067 |
|  | X - 24970 |  |  | 0.0106 | 0.4193 | 0.0895 | 0.7491 | 0.0128 | 0.30119 | -0.02152 | 0.6239 | 0.0673 |
| ^1^Beta coefficients were obtained from elastic net regression in the testing set. A total of 893 participants were randomized to either the training set (n=625) or the testing set (n=268) in a 7 to 3 fashion. | | | | | | | | | | | | |
| ^2^Linear regression was conducted in the overall sample (n=893). Multivariable linear regression models were adjusted for age, sex, season, total energy intake, leisure-time physical activity, alcohol consumption, smoking status, education, family history of diabetes, use of lipid-lowering drugs, hypertension at baseline, history of cardiovascular disease, history of cancer, fiber, vegetable and fruits, meat, soft drinks, coffee, and body mass index as well as baseline diabetes. The Bonferroni corrected *P* value was considered statistically significant. | | | | | | | | | | | | |
|  |  |  |  |  |  |  |  |  |  |  |  |  |
| The texts in bold indicate statistical significance in multivariable linear regression models after multiple corrections (*P*<0.05/46). | | | | | | | | | | | | |

**The analytic codes for main analyses**

/*Cox regression model*/

**proc** **phreg** data=dataset;

class group (param=reference ref=first);

model time*status(**0**)=exposure covariate/risklimits;

**run**;
